# Comparative transcriptomic profiling of peach and nectarine cultivars reveals cultivar-specific responses to chilled postharvest storage

**DOI:** 10.3389/fpls.2022.1062194

**Published:** 2022-11-25

**Authors:** Antonella Muto, Leonardo Bruno, Maria Letizia Madeo, Richard Ludlow, Michele Ferrari, Louise Stimpson, Claudio LoGiudice, Ernesto Picardi, Antonio Ferrante, Luisa Pasti, Carsten T. Müller, Adriana Ada Ceverista Chiappetta, Hilary J. Rogers, Maria Beatrice Bitonti, Natasha Damiana Spadafora

**Affiliations:** ^1^ Department of Biology, Ecology and Earth Sciences, University of Calabria, Cosenza, Italy; ^2^ School of Biosciences, Cardiff University, Cardiff, United Kingdom; ^3^ Department of Biosciences, Biotechnology and Biopharmaceutics, University of Bari, Bari, Italy; ^4^ Institute of Biomembranes, Bioenergetics and Molecular Biotechnologies, Consiglio Nazionale delle Ricerche, Bari, Italy; ^5^ Department of Agricultural and Environmental Science, Università degli Studi di Milano, Milan, Italy; ^6^ Department of Environment and Prevention Sciences, University of Ferrara, Ferrara, Italy; ^7^ Department of Chemical, Pharmaceutical and Agricultural Sciences, University of Ferrara, Ferrara, Italy

**Keywords:** transcriptome, postharvest, ERF transcription factors, ethylene, *Prunus*

## Abstract

**Introduction:**

Peach (*Prunus persica* (L.) Batsch,) and nectarine fruits (Prunus persica (L.) Batsch, var nectarine), are characterized by a rapid deterioration at room temperature. Therefore, cold storage is widely used to delay fruit post-harvest ripening and extend fruit commercial life. Physiological disorders, collectively known as chilling injury, can develop typically after 3 weeks of low-temperature storage and affect fruit quality.

**Methods:**

A comparative transcriptomic analysis was performed to identify regulatory pathways that develop before chilling injury symptoms are detectable using next generation sequencing on the fruits of two contrasting cultivars, one peach (Sagittaria) and one nectarine, (Big Top), over 14 days of postharvest cold storage.

**Results:**

There was a progressive increase in the number of differentially expressed genes between time points (DEGs) in both cultivars. More (1264) time point DEGs were identified in ‘Big Top’ compared to ‘Sagittaria’ (746 DEGs). Both cultivars showed a downregulation of pathways related to photosynthesis, and an upregulation of pathways related to amino sugars, nucleotide sugar metabolism and plant hormone signal transduction with ethylene pathways being most affected. Expression patterns of ethylene related genes (including biosynthesis, signaling and ERF transcription factors) correlated with genes involved in cell wall modification, membrane composition, pathogen and stress response, which are all involved later during storage in development of chilling injury.

**Discussion:**

Overall, the results show that common pathways are activated in the fruit of ‘Big Top’ nectarine and ‘Sagittaria’ peach in response to cold storage but include also differences that are cultivar-specific responses.

## Introduction

Peach (*Prunus persica* (L.) Batsch) is one of the most commercially important fruit species from the Rosaceae family. Since its first cultivation in ancient China 3000 years ago, many peach cultivars have been developed including nectarines (*Prunus persica* (L.) Batsch, var nectarine), particularly appreciated by consumers due to their hairless skin, as well as their aroma and flavour (Wen et al., 1995). Peach cultivation is limited geographically to warmer regions, with China, Italy, Greece and Spain accounting for three quarters of worldwide production (United Nations Food and Agriculture Organization Statistics Division (FAOSTAT), 2019) (http://www.fao.org/faostat/en/#data/QC).

Non-producing countries import large quantities of peaches from a range of worldwide producers. Loss through spoilage is a serious risk for perishable foodstuffs, still representing the second highest cause of food waste (Lipińska et al., 2019). Long-distance shipping is slow and low-temperature storage (0-5°C), is commonly used to minimize spoilage, extending the maximum storage period to around 14-21 days (Aubert et al., 2014). However, longer storage periods of over 3 weeks frequently result in chilling injury (CI, Lurie and Crisosto, 2005), causing severe commercial postharvest loss, second only to fungal-related decay (Crisosto and Valero, 2008).

During CI, fruit of sensitive species exposed to low temperature undergo a range of molecular, biochemical and physiological changes which cause metabolic disorders resulting from crosstalk between ripening and senescence processes at the physiological, biochemical, cellular and molecular levels (Parkin et al., 1989; Sevillano et al., 2009). This results in a suite of spoilage symptoms including surface pitting, internal breakdown such as mealy or woolly texture, leathery fruit with a hard texture and minimal juice, internal browning/discoloration, failure to ripen, growth inhibition, wilting, loss of flavour, and decay.

A mealy texture has been associated with defective cell wall disassembly and internal breakdown of tissue caused by changes in pectin methylesterase (PME) levels, although there is inconsistency in this association, with both a reduction and increase in PME levels being reported (Brummell et al., 2004; Jin et al., 2009). This deregulation leads to reduced levels of methoxyl pectins and accumulation of unesterified pectins (Lurie and Crisosto, 2005). These form a gel structure that captures free water from the flesh, resulting in chilling induced mealiness (Zhou et al., 2000; Crisosto and Valero, 2008). An increase in endo-1,4-glucanase (Endo-14G) and decrease in expansin (Exp) activity has also been associated with the mealiness. Leatheriness has been linked to cell wall thickening and a reduction in the activity of cell wall modifying enzymes, including polygalacturonase (PG) and β-galactosidase, as well as a downregulation of the ethylene synthesis pathway (Lurie and Crisosto, 2005). In contrast, internal browning is likely a senescence-based phenotype, with a change in membrane permeability resulting in oxidation of phenolics by polyphenol oxidase (Brummell et al., 2004; Lurie and Crisosto, 2005). Membrane fluidity is also changed upon cold exposure mainly through the modulation of genes and enzymes involved in the metabolism of lipid components, and this also plays a role in internal browning (Routaboul et al., 2000; Browse and Xin, 2001; Martz et al., 2006).

At a transcriptional level cold resistance in peach fruit is linked to changes in redox metabolism, stress-responsive genes (Tanou et al., 2017; Lurie, 2021). In particular, ethylene and auxin pathways have been identified as particularly important in the development of CI symptoms (Vizoso et al., 2009; Puig et al., 2015). *ERF* genes, belonging to the AP2/EREBP multigene family, mediate a positive effect of ethylene on CI-related internal browning (Wang et al., 2017; Lurie, 2021). A total of 32 *ERF* genes changed in expression in response to chilled storage for three weeks (Wang et al., 2017). Several *ERF*s were co-expressed with both cell wall related and lipid metabolism genes, with indications that the same ERFs may control both pathways.

According to Zhang et al. (2012) the *AP2/E*RF multigene family in peach includes 131 members, with the *ERF* genes divided into 11 groups. This was based on the classification made previously in Arabidopsis (Nakano et al., 2006), although with a new annotation of the peach genome (Verde et al., 2017) this classification needs re-evaluating. The *AP2/EREBP* superfamily is divided into three subfamilies: *AP2*, *ERF* and *RAV*. The AP2 subfamily, which includes AP2 and ANT (AINTEGUMENTA) subgroups, is characterized by two AP2 domains. The ERF subfamily is characterized by a single AP2 domain and includes ERF (Ethylene Responsive Factor) and DREB (Dehydration-Responsive Element-Binding Protein) groups based on the type of amino acid residues. The first binds to the AGCCGCC element, the GCC box (Ohme-Takagi and Shinshi, 1995; Hao et al., 1998), while the second bind the dehydration response element (TACCGACAT) (Jiang et al., 1996). Both ERF and DREB subfamily proteins are involved in both abiotic (Zhang et al., 2009) and biotic (Guo et al., 2014) stress responses (Hong and Kim, 2005; Ito et al., 2006; Fang et al., 2015). Lastly, the RAV subfamily members contain a B3-like domain in their N-terminal region, in addition to an AP2-domain in their C-terminal region (Swaminathan et al., 2008). This class of genes is involved in the regulation of gene expression in response to phytohormones such as ethylene and brassinosteroids, as well as in response to biotic and abiotic stresses (Mittal et al., 2014).

In fruit, it has been proposed that ethylene is involved in upregulating ERFs, ERF targets include genes involved in the modification of cell wall structure (González-Agüero et al., 2008; Vizoso et al., 2009) leading to rapid flesh softening. However, the gene regulatory networks and transcription factors involved in cold stress responses in fruit are not fully understood (Lurie, 2021).

Here, we examined separately the effects of postharvest cold storage during a time-course on fruits of two cultivars: an early ripening peach (cv. Sagittaria) and a midseason ripening nectarine (cv. Big Top) grown in the same Calabrian farm. Both ‘Big Top’ and ‘Sagittaria’ respond well to chilled storage (Muto et al., 2022) although ‘Sagittaria’ is less sweet, more acid, less juicy and more bitter and more astringent after 7 days of storage, indicating some differences in their physiology. The aim here was to elucidate the early molecular events occurring in peach fruit during cold storage before CI develops. We show very early differences in global response between the two cultivars, but also shared responses involving hormone signaling, and we examine co-expression of ethylene signaling with genes known to be later involved in CI.

## Materials and methods

### Plant material, chilling treatment and physiological measurements

Two cultivars of *Prunus persica* (L), Batsch were used in the study: cv. Sagittaria (SAG), an early-ripening peach, and cv. Big Top (BT), a medium late-ripening nectarine, both yellow melting flesh type. Both cultivars were grown at the “Campo Verde” Agricultural Company, Calabria, Italy [(39°48′58″ N, 16°12’06” E, 382 meters above sea level, (masl)]. Sampling was carried out in the 2017 and 2018 summer seasons. Commercially mature fruit was collected manually at the time of commercial harvest and for each cultivar, 50 kg of fruit (about 300 fruit), were selected for uniformity in size, maturity, appearance and lack of defects and then transported to the laboratory. Fruits were sampled before storage (Day 0), and cold stored at 1°C for 1 (Day 1), 5 (Day 5), 7 (Day 7) and 14 (Day 14) days, (100 peaches for each time point) after storage fruits were transferred to a growth chamber at 22°C for 36 h acclimatisation. For each time point three biological replicates were considered. To verify the assessment of maturity stage, 15 fruits (5 fruits for each biological replicate) considered of equal maturity based on appearance, were tested for flesh firmness (N) and total soluble solids (Brix) content (SSC %; **Supplementary Table 1**). Total soluble solids were measured using an optical refractometer MA871 (Milwaukee, Rocky Mount, NC, USA). Titratable acidity was measured as previously described (Muto et al., 2022). Firmess was measured using a penetrometer as described previously (Muto et al., 2020). At each sampling time, slices of mesocarp (about 1 cm thick) were combined, frozen in liquid nitrogen and stored at -80°C for further use. For the transcriptome (2017 season) and molecular analyses, three biological replicates of five fruit each were used.

Ethylene was collected from individual fruits in 300 ml jars after 30 mins equilibration at each storage timepoint. Headspace (1 ml) was removed from the jar and injected into a 6890N gas chromatograph (Agilent, Santa Clara, CA, USA) operated isothermally at 100°C with a constant flow rate of 2 ml min ^-1^ helium. The GC inlet was operated at 200°C and compounds were separated with a splitless gas flow on an Rt-Alumina BOND/KCl column (30m, 0.53 mm ID, 10 µm film thickness, Restek, Ripley, UK). The FID was operated at 200°C with 40 mL min ^-1^ flow rate of hydrogen, 450 mL min^-1^ compressed air, and 45 mL min^-1^ nitrogen. Ethylene was quantified against a standard curve (10 -100 ppm) using a 100 ppm ethylene standard (Thames Restek, High Wycombe, UK). Peaches were weighed and data expressed as ppm/g fresh weight/h.

### RNA isolation and RNA-seq library synthesis

Total RNA extraction was performed separately for each of the 30 samples (three biological replicates of five fruit each were used for each five stages analysed for SAG and BT) using 100mg of the peach mesocarp powder according to the manufacturer’s recommendations using the Agilent Total RNA Isolation Mini Kit (Agilent Technologies, Santa Clara, CA, USA).

Cytoplasmatic rRNA removal was performed for each total RNA sample using the Ribo-Zero rRNA Removal Kit (Epicentre, Madison, WI, USA) and rRNA-depleted RNA was used to prepare thirty mRNA seq-strand oriented libraries using the TruSeq Stranded Total RNA Sample Prep Kit (Illumina, San Diego, CA, USA), according to the manufacturer’s instructions. After quality checking of the prepared libraries using a Qubit 2.0 Fluorometer (Invitrogen, Carlsbad, CA) and an Agilent 2100 Bioanalyzer, cDNAs were processed by IGA Technology Services using a HiSeq2500 sequencing platform (Illumina, San Diego, CA) to generate pair-end reads of 125 bp for each fragment. The raw data are available at the SRA BioProject PRJNA798864.

### RNA-Seq quality control and preprocessing and reference genome-based reads mapping

RNA-Seq reads in FASTQ format were inspected using the FASTQC program (http://www.bioinformatics.babraham.ac.uk/projects/fastqc/). Adaptors and low-quality regions (phred cut-off 20) were trimmed using fastp tools (https://github.com/OpenGene/fastp), excluding reads with a final length of less than 50 bases. Cleaned reads were subsequently aligned onto the peach reference genome v2.0 (Verde et al., 2017) using the STAR read aligner (https://code.google.com/p/rna-star/) (Dobin et al., 2013). Mapped reads in SAM format were converted to the binary BAM format using SAMtools (http://www.htslib.org/doc/samtools.html) (Li et al., 2009).

### Differential analysis

Genes with count value showing 0 in >20% samples were filtered and 15,975 genes for SAG peach and 16,096 genes for BT nectarine were obtained for further analysis. Differentially expressed genes between time points (DEGs) were identified using the DESeq2 package in R. The threshold for differentially expressed (DE) genes was set to a fold-change of 1.5 and a P­ value adjusted ≤ 0.05. The R package ImpulseDE2 (Fischer et al., 2018) was used to identify genes differentially expressed during the time course. This program, specifically designed for time course data, distinguishes genes whose expression is consistently up- or down-regulated throughout the time course (MONOTONOUS INCREASE (MI) and DECREASE (MD) genes), from transiently up- or down-regulated [TRANSIENT INCREASE (TI) and DECREASE (TD)] genes. This method is based on a negative binomial noise model with dispersion trend smoothing by DESeq2 and uses the impulse model to constrain the mean expression trajectory of each gene.

### Bioinformatic tools, enrichment analysis and weighted gene co-expression network analysis

Transcription factors (TFs) were identified using the classification described in the PlnTFDB database (http://plntfdb.bio.uni‐potsdam.de/) (Pérez-Rodríguez et al., 2010). Heat maps and PCA (Principal Component Analysis (PCA) were both created using the ClusVis web tool (Mao et al., 2005; Metsalu and Vilo, 2015). Bubble charts were constructed using R version 3.5.0. GO categorization results were expressed as three independent hierarchies for molecular function, biological process, and cellular component, using AgriGO software (http://systemsbiology.cau.edu.cn/agriGOv2/) for the statistical analyses of the gene ontology data, and KOBAS 3.0 software (http://kobas.cbi.pku.edu.cn/) to test the statistical enrichment of differentially expressed genes in KEGG (http://www.genome.jp/kegg/) pathways (Mao et al., 2005).

Co-expressed DEGs were identified by a scale-free weighted gene correlation network analysis (WGCNA; Pearson**’**s correlation coefficient ≥ 0.8 and p ≤ 0.05), with a soft-thresholding power 16 (Langfelder and Horvath, 2008; Fischer et al., 2018). Separate analyses were completed for each cultivar. Module-trait relationship figures were created using RStudio. Analysis of the 746 SAG DEGs and 1264 BT DEGs compared each time point to the next (i.e., Day 1 vs Day 0, Day 5 vs Day 1, Day 7 vs Day 5, Day 14 vs Day 7) producing co-expression modules designated by a colour, and consisting of genes with similar expression patterns over time (trait **‘**DAY**’**).

### Promoter and phylogenetic analysis

Promoter analysis was performed using the PWM scan tool (Ambrosini et al., 2018). All *Prunus persica* promoter sequences were scanned for ERF binding cis-elements to identify genes that could potentially respond to the corresponding TFs. The TF binding motifs available to scan for in the promoter sequences were sourced from the JASPAR CORE 2018 Plants motif library (Khan et al., 2018). Promoter analysis data was then combined with the Impulse data (Fischer et al., 2018) to identify DEGs that also potentially respond to ERF TFs.

### Motif display and phylogenetic analysis of predicted AP2/ERF proteins in peach.

The online web tool MEME (version 4.8.1) was used to search the conserved motifs shared by peach AP2/ERF proteins (http://meme.nbcr.net/meme/cgi-bin/meme.cgi (Bailey et al., 2006). Parameters were set as described by Tong et al. (2009) and Wang et al. (2010) and used previously in Zhang et al. (2012) in the analysis of peach AP2/ERF genes: 0 or 1 occurrence of a single motif per sequence, motif width ranges of 10 to 300 amino acids, and 5 as the maximum number of motifs that must be found. All other parameters were set at default. The amino acid sequences of the AP2/ERF superfamily in Arabidopsis and peach, [from the Plant Transcription Factor Database (PTFD)] were aligned using MUSCLE to create a phylogenetic tree [Neighbour-Joining, 1000 bootstrap, Poisson correction method, and gamma distribution (shape parameter =1)] https://blast.ncbi.nlm.nih.gov/Blast.cgi) (Altschul and Lipman, 1990; Edgar, 2004). Evolutionary analyses were conducted in MEGA7 (Kumar et al., 2016).

### qRT-PCR

Total RNA (1µg) from each sample was retro-transcribed into cDNA using an iScript™ cDNA synthesis kit (Invitrogen), according to the manufacturer’s protocol. Gene expression analyses were carried out on a STEP ONE instrument (Applied Biosystems, Monza, Italy) using Power SYBR Green PCR Master Mix 2X (Applied Biosystem, Monza, Italy). Amplification reactions were prepared in a final volume of 20 µl by adding 10 µl Power SYBR^®^ Green PCR Master Mix (Applied Biosystems), 2 µl of cDNA (40 ng) and 1 µl each primer (0.2 µM). All reactions were run in triplicate. Melting curve analysis was also performed. The cycling parameters were as described in Muto et al. (2020). *PpTEF2* was used as an internal control to normalize small differences in template amounts according to Tong et al. (2009).

The primer sets used are listed in **Supplementary Table 2**: four genes related to ethylene and auxin pathways were selected from the literature and the PpTEF2 gene (encoding for translation elongation factor 2) was used as an internal control to normalise small differences in template amounts according to Tong et al. (2009). Only for one gene (PRUPE_3G062800), specific primer pairs were designed using Primer3 (Kõressaar and Remm, 2007; Untergasser et al., 2012; Kõressaar et al., 2018) and then specificity checked with Primer-BLAST (Ye et al., 2012) against “Prunus persica (taxid:3760)”. Primer efficiency was calculated from a standard curve analysis with a dilution series from 1:10, to 1:800 using the formula E = 10-1/slope. The efficiency of the primers for PRUPE_3G062800 gene was 98%. Relative quantification of gene expression was calculated according to Schmittgen and Livak (2008). Statistical analyses were performed on ΔCt values, first checking for deviations from normality (Kolmogorov-Smirnov test) and tested for homogeneity (Leven Median test) and then analysed by ANOVA and a Tukey’s rank test (P < 0.05).

## Results

### ‘Sagittaria’ and ‘Big Top’ differ in physiology over cold storage

**Figure 1:**
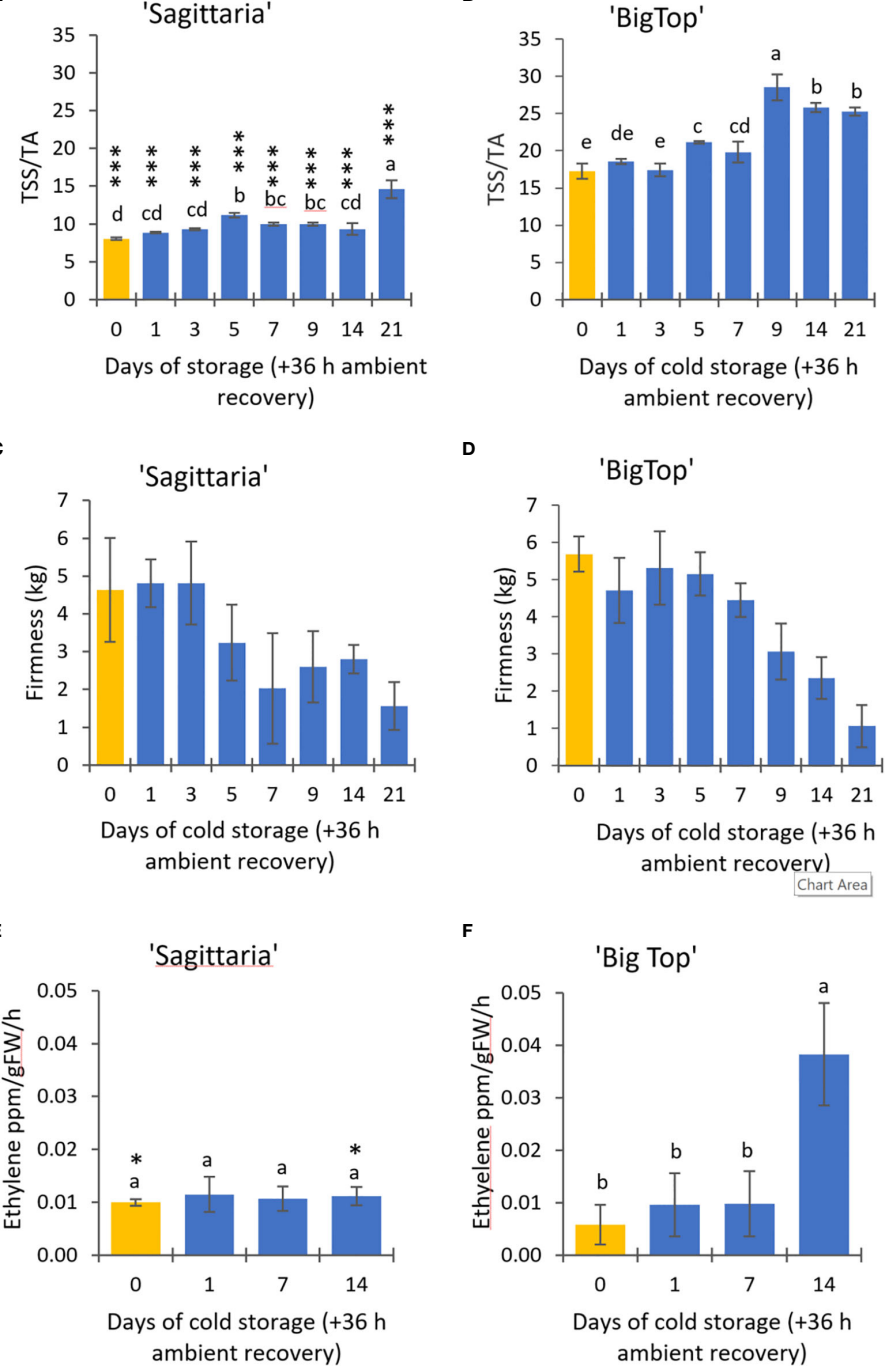
Effects of cold storage on ‘Sagittaria’ peaches and ‘BigTop’ nectarines at 1°C followed by 36 h recovery at ambient temperature (22 °C). **(A, B)** maturity index (total soluble solids/titratable acidity) (n=3); **(C, D)** firmness (kg) (n=10). **(E, F)** ethylene emission (n=6). Mean ± SD; letters indicate significant differences between time points based on ANOVA and Tukey’s rank test (P < 0.05). Asterisks indicate significant differences between ‘Sagittaria’ and ‘Big Top’ values for each day (*** <0.001, * < 0.05).

Maturity index (assessed as the total soluble solids divided by the titratable acidity) was significantly lower in in the peach,’Sagittaria’ (SAG), than the nectarine, ‘Big Top’ (BT) fruit at all timepoints (**Figure 1A, B**). It remained relatively constant in SAG throughout 14 days of sampling, however after 21 days it rose significantly. In BT, in contrast, it peaked after 9 days and thereafter fell back slightly remaining constant even after 21 days of cold storage. Firmness fell in both cultivars (**Figures 1C, D**) and was only significantly different between the cultivars at harvest and after 5 and 7 days of storage. Ethylene emission between the two cultivars showed different profiles (**Figure 1E, F**): in the peach,’Sagittaria’ (SAG), more ethylene was produced at harvest but thereafter remained constant. In the nectarine, ‘Big Top’ (BT), ethylene production rose significantly (P < 0.05) on day 14 and was significantly higher than from SAG fruit at this timepoint. Neither cultivar developed any visual signs of CI in the fruit during the storage period + 36 h recovery at 22°C (**Supplementary Figure 1**) although by 21 d of cold storage they were considered non-marketable by the producer (pers. comm.) due to the reduced firmness.

### Most genes that changed in expression with cold storage (DEGs) were identified in earlier storage timepoints

**Figure 2:**
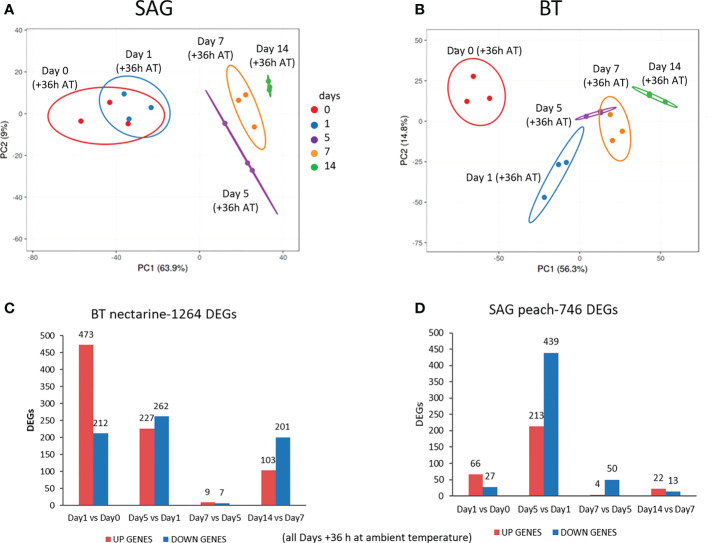
Gene expression changes during cold storage. Principal Component Analysis (PCA) of normalised raw gene expression counts in SAG peach **(A)** and BT nectarine **(B)** for each day of cold storage at 1 °C (+36 h at ambient temperature (AT) 22 °C). Differentially expressed genes (DEGs) among successive storage time points for each cultivar: Sagittaria (SAG) peach **(C)** and Big Top (BT) nectarine **(D)**. Gene expression level values were normalized by the DESEQ2 software (pvalue corrected < 0.05 andlog2FC> 1.5).

To assess reproducibility and similarity across the datasets, principal component analysis (PCA) based on normalised raw gene expression counts calculated by DESeq2, was applied. Close grouping of the three biological replicates indicates good repeatability (**Figure 2A, B**). In both cultivars, samples separated along PC1 with some overlap between time points. In SAG, PC1 (x-axis) and PC2 (y-axis) explained 63.9% and 9% of the total variance, respectively (**Figure 2A**). In SAG, Day 0 and Day 1 overlapped while time point Day 5, Day 7 and Day 14 were well separated along PC1 (**Figure 2A**). In BT (**Figure 2B**) PC1 and PC2, explained 56.3% and 14.8% of the total variance, respectively. In BT, Day 0 and Day 1 separated while there was an overlap in the profiles between the Day 5 and Day 7 time points. Furthermore, a clear separation was found between Day 7 and Day 14 time points.

More storage time DEGs were detected in BT nectarine (1264) compared to SAG peach (746), respectively (**Figure 2C, D**) and the two cultivars also differed in the time points that showed the greatest changes in expression. In SAG there were only 93 DEGs in the Day 1 vs. Day 0 comparison whereas most were detected between Day 5 vs. Day 1 (652) (**Figure 2C**). Thereafter the number of DEGs drastically decreased to 54 between Day 7 vs. Day 5 and 35 in the Day 14 vs. Day 7 comparison. A different pattern was observed in BT (**Figure 2D**), which exhibited the highest DEG number in the Day 1 vs. Day 0 and Day 5 vs. Day 1 comparisons, (685 and 489 respectively). As for SAG, there was then a drastic reduction in the Day 7 vs. Day 5 comparison with only 16 DEGs, but unlike SAG, more DEGs (304) were then seen in the Day 14 vs. T7 comparison. In both cultivars, more DEGs were up-regulated than down-regulated in Day 1 vs. Day 0 but the pattern was reversed in Day 5 vs. Day 1, and again more DEGs were down-regulated in the Day 14 vs. Day 7 comparison for BT where substantial numbers of DEGs were seen.

### Time course of differential gene expression in the two cultivars

**Figure 3:**
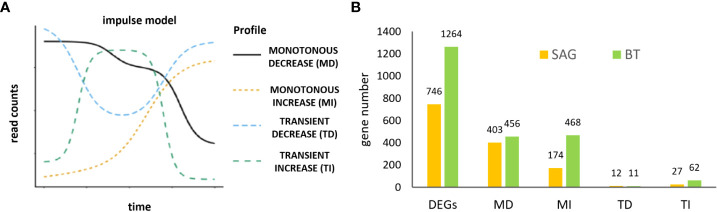
Expression profiling of time course transcriptome data using ImpulseDE2 (Fischer et al., 2018) to identify clusters of genes that are continuously up- or down-regulated MONOTONOUS INCREASE (MI) and DECREASE (MD) genes and transiently up- or down-regulated, TRANSIENT INCREASE (TI) and DECREASE (TD) **(A)**; numbers of differentially expressed genes (DEGs) in the MI, MD, TI and TD clusters for SAG peach and BT nectarine **(B)**.

Based on ImpulseDE2 analysis, the DEGs were classified into four distinct expression clusters (**Figure 3A**): Cluster MD (monotonous down, MD), included genes consistently decreasing in expression through the time course, comprising 403 genes in SAG and 456 genes in BT. Cluster MI (monotonous increase, MI), comprised genes with a continuous increase through the storage time course with 174 genes in SAG and 468 genes in BT. Clusters TD (transiently down, TD) and TI (transiently increased, TI) comprised genes with fluctuating expression. Cluster TD genes decreased in expression transiently and comprised 12 genes in SAG peach and 11 in BT nectarine; Cluster TI were transiently up-regulated consisting of 27 genes in SAG peach and 62 genes in BT nectarine. (**Figure 3B**). Thus, a common trend was observed in the two cultivars consisting in a prevalence of continuously down- (MD) or up-(MI) regulated expression.

**Figure 4:**
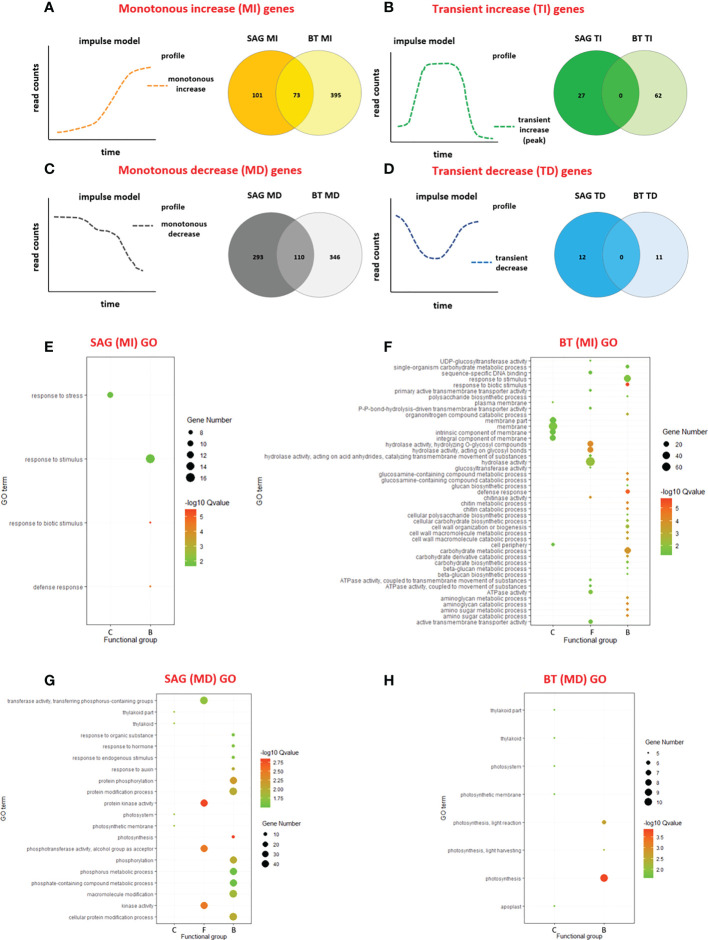
Differential expression in the two cultivars. Venn diagrams of shared and unique genes related to temporal profiles in the two cultivars, in the four different clusters MONOTONOUS INCREASE (MI) **(A)**, MONOTONOUS DECREASE (MD) **(B)**, TRANSIENT INCREASE (TI) **(C)** and TRANSIENT DECREASE (TD) **(D)**. GO enrichment analysis of MI and MD cluster genes in the two cultivars: MI genes in SAG (174) **(E)**, and BT (468) **(F)**; MD cluster genes in SAG (403) **(G)** and BT (456) **(H)**, including C, cellular components; F, molecular functions and B, biological processes. The pathway enrichment analysis was performed with KOBASS, online tool, and the detailed information is presented as a bubble chart. The size of the bubbles represents the number of assigned genes, and the color of bubbles represents the -log10 (Q-value).

Gene identities, however, differed between the two cultivars for each expression cluster. Amongst the MI DEGs, in both cultivars the majority of the genes were unique to each cultivar: 101 (58%) in SAG peach and 395 (84%) in BT nectarine, while only 73 were common to both cultivars (**Figure 4A**). A similar pattern was seen in the MD DEGs with more similar numbers of cultivar specific DEGs: 293 (72%) in SAG peach and 346 (76%) in BT nectarine, while 110 DEG were common to both cultivars (**Figure 4B**). In the two transiently expressed groups of genes, a higher number of TI genes were found in the BT, with similar numbers of genes for both cultivars in the TD cluster. None of either TI or TD genes was common to the two cultivars (**Figure 4C, D**).

### Gene enrichment analysis of monotone and transient clusters

In the MI cluster of SAG peach, only four biological process subcategories were significantly enriched by Gene Ontology (GO) term enrichment (P < 0.05): the most significantly enriched related to response to biotic stimulus and defence response (**Figure 4E**). A higher number (45) of GO subcategories were significantly enriched (P < 0.05) in the MI cluster of BT nectarine. Again, response to biotic stimulus and defence response were significantly enriched, as were also cell wall and carbohydrate metabolism, chitinase metabolism and amino sugar metabolism (**Figure 4F**) Figure. The opposite trend was seen for the MD cluster of genes, where more GO categories were significantly enriched in SAG (20) compared to BT (8; **Figure 4G**). Most significantly enriched in SAG were kinase, protein kinase and phosphorylase activities, as well as protein modification, photosynthesis, phosphotransferase activity, and response to auxin (P < 0.05). In contrast, for the MD cluster of BT, the most significantly enriched processes were related to photosynthesis (**Figure 4H**). Interestingly, the TI cluster from SAG showed a similar trend to the MI cluster (**Supplementary Figure 2A**) with the most significant enrichment of response to biotic stimulus and defence response, while only ADP binding was significantly enriched in the TI cluster of BT (**Supplementary Figure 2B**) and no significantly enriched GO categories were found for the TD genes of either peach cultivar.

KEGG enrichment analysis (**Supplementary Figure 3A-D**) revealed both similarities and differences in the pathways affected in the two cultivars. Downregulation of photosynthesis was common to both cultivars in the MD cluster genes, while several metabolic pathways were upregulated in the BT but not the SAG MI cluster. Hormone signal transduction was significantly upregulated in BT, while both up- and downregulation was observed in SAG. Sesquiterpenoid and triterpenoid biosynthesis as well as folate biosynthesis were enriched in the TI cluster of SAG (**Supplementary Figure 4A**), while starch and sucrose metabolism was the only pathway enriched in the TI cluster of BT. No significant enriched pathways were found for the TD genes for either cultivar.

### Hormone signalling modulation during the time course

In both cultivars, the majority of differentially expressed monotone genes related to hormone biosynthesis and signalling involved ethylene (21 genes; **Table 1**) and auxin (20 genes; **Table 2**). Three of the ethylene-related genes were related to ethylene biosynthesis: one *PpACS* gene and two *PpACO* genes. The *PpACS* gene was upregulated in both SAG and BT, whereas *ACO* gene expression patterns differed, one being upregulated in BT and the other downregulated in SAG. Of the genes related to hormone perception/signalling, the ethylene receptor genes *PpETR2* and *ppEBF1* were upregulated only in BT, while a MAPKKK-related gene was downregulated only in SAG, and *PpEIN3* was down-regulated only in BT (**Table 1**).

**Table 1:** Expression in transcriptome of monotone genes related to ethylene biosynthesis and signaling.

DESCRIPTION	ID	MI_SAG	MI_BT	MD_SAG	MD_BT
mitogen-activated protein kinase kinase kinase (MAPKKK)-related	PRUPE_1G447700				
1-aminocyclopropane-1-carboxylate oxidase (ACO)*	PRUPE_3G209900				
ethylene receptors 2 (ETR2)	PRUPE_1G034300				
EIN3	PRUPE_2G070300				
EBF1/EIN3-binding F box protein 1	PRUPE_7G244300				
1-aminocyclopropane-1-carboxylate synthase (ACS)	PRUPE_2G176900				
1-aminocyclopropane-1-carboxylate oxidase (ACO)	PRUPE_2G251400				
ethylene response factor 1 (ERF)	PRUPE_1G037900				
ethylene response factor 1 (ERF)	PRUPE_1G214900				
ethylene response factor 1 (ERF)	PRUPE_6G064700				
ethylene response factor 1 (ERF)	PRUPE_3G240000				
ethylene response factor 1 (ERF)*	PRUPE_2G272500				
ethylene response factor 1 (ERF)	PRUPE_6G039700				
ethylene response factor 1 (ERF)	PRUPE_5G090800				
ethylene response factor 1 (ERF)	PRUPE_2G272300				
ethylene response factor 1 (ERF)	PRUPE_2G289500				
ethylene response factor 1 (ERF)	PRUPE_4G051200				
ethylene response factor 1 (ERF)	PRUPE_4G051400				
ethylene response factor 1 (ERF)	PRUPE_7G194400				
ethylene response factor 1 (ERF)*	PRUPE_3G062800				
ethylene response factor 1 (ERF)	PRUPE_8G224600				

**Figure 5:**
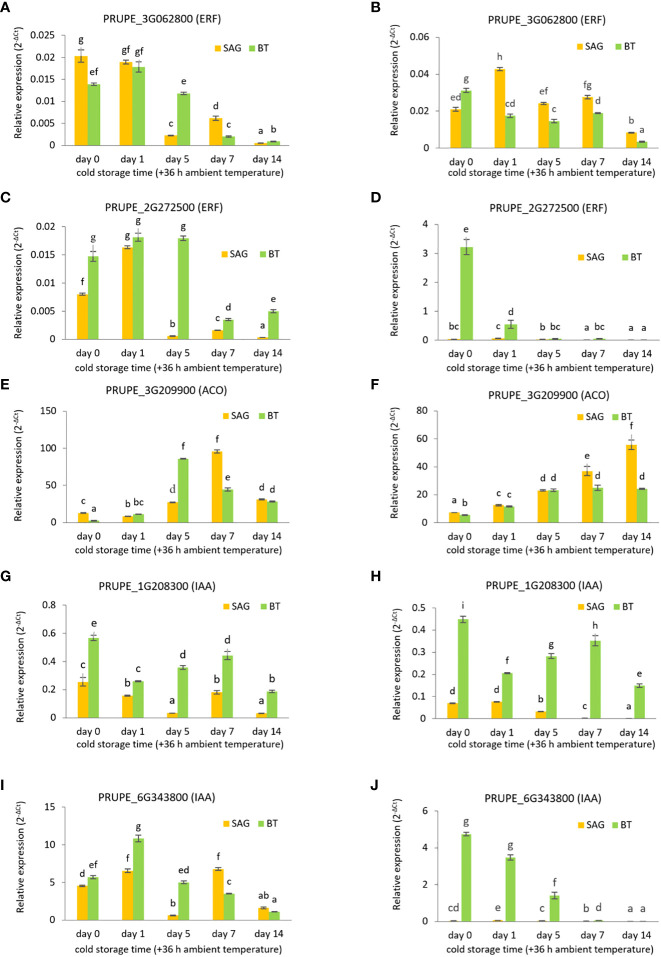
Real-time PCR analysis of selected DEGs related to ethylene and auxin signalling in SAG peach and BT nectarine, during cold storage treatment (1°C) at day 0, 1, 5, 7 and 14 followed by 36 h recovery at ambient temperature (22 °C). in two seasons 2017 **(A, C, E, G, I)** and 2018 **(B, D, F, H, J)**. PRUPE_3G062800 (*ERF*) **(A, B)**; PRUPE_2G272500 (*ERF*) **(C, D)**; PRUPE_3G209900 (*ACO*) **(E, F)**; PRUPE_1G208300 (*AUX/IAA*) **(G, H)**; PRUPE_6G343800 (*AUX/IAA*) **(I, J)**. Different letters indicate significant differences among cultivars considering all time points and both years. Statistical analyses were performed using ANOVA and Tukey’s ranked test (P < 0.05). Data are the mean ± SE; n=3.

*unigenes analysed by qRT-PCR (Section 3.6 and **Figure 5**)Red indicates up-regulated and blue indicates down-regulated.

**Table 2:** Expression of monotone genes related to auxin biosynthesis and signaling.

DESCRIPTION	ID	MI_SAG	MI_BT	MD_SAG	MD_BT
indole-3-pyruvate monooxygenase EV-COMP (YUC)	PRUPE_6G157400				
indole-3-pyruvate monooxygenase EV-COMP (YUC)	PRUPE_6G157500				
indole-3-pyruvate monooxygenase EV-COMP (YUC)	PRUPE_8G252500				
auxin-responsive Gretchen Hagen3(GH3)	PRUPE_6G226100				
small auxin upregulated RNA (SAUR)	PRUPE_2G317100				
small auxin upregulated RNA (SAUR)	PRUPE_7G167000				
small auxin upregulated RNA (SAUR)	PRUPE_7G192600				
small auxin upregulated RNA (SAUR)	PRUPE_8G081100				
small auxin upregulated RNA (SAUR)	PRUPE_8G081700				
small auxin upregulated RNA (SAUR)	PRUPE_8G081800				
small auxin upregulated RNA (SAUR)	PRUPE_8G081900				
small auxin upregulated RNA (SAUR)	PRUPE_8G082100				
small auxin upregulated RNA (SAUR)	PRUPE_8G081300				
small auxin upregulated RNA (SAUR)	PRUPE_8G157800				
small auxin upregulated RNA (SAUR)	PRUPE_8G157900				
auxin/indole-3-acetic acid (AUX/IAA protein)	PRUPE_7G234800				
auxin/indole-3-acetic acid (AUX/IAA protein)	PRUPE_7G247500				
auxin/indole-3-acetic acid (AUX/IAA protein)*	PRUPE_1G208300				
auxin/indole-3-acetic acid (AUX/IAA protein)*	PRUPE_6G343800				
Dormancy auxin associated	PRUPE_6G319600				

*unigenes analysed by qRT-PCR (Section 3.6 and **Figure 5**)Red indicates up-regulated and blue indicates down-regulated.

The two cultivars also differed in the expression of their *ERF* transcription factors. A total of 14 *ERF*s were identified but only three were represented in the SAG peach MD cluster whereas twelve were found in the BT MI or MD nectarine clusters. The pattern of expression of the 14 *ERF* genes also differed between the two cultivars. Except for the *ERF* PRUPE_3G062800 (MD), none of them showed the same expression pattern in both cultivars but 7/12 BT and all the SAG *ERF*s were downregulated (**Table 1**).

Three indole-3-pyruvate monooxygenases (*YUC*) genes involved in auxin biosynthesis were differentially modulated in the two cultivars (**Table 2**): two upregulated exclusively in BT and one downregulated in both cultivars. Other genes were mainly related to auxin early response genes: eleven small auxin upregulated RNA (*SAUR*) genes were represented. In BT these were either up-or downregulated, while in SAG more were down- than upregulated. Two were down-regulated in both cultivars: PRUPE_8G081100 and PRUPE_8G082100 (**Table 2**). Of the four auxin/indole-3-acetic acid (*AUX/IAA*) genes represented in the MI and MD clusters, none were upregulated in SAG, while two were downregulated, and in BT two were up and one was downregulated. One *AUX/IAA* gene (PRUPE_6G343800) was down-regulated in both cultivars. However, an auxin-responsive Gretchen Hagen3 (*GH3*) family auxin homeostasis modulator was only upregulated in SAG.

### Verification of the transcriptome analysis by real-time quantitative PCR

Given the important role of both hormones in modulating fruit cold responses, expression of five ethylene- and auxin-related DEGs were validated through qRT-PCR over two seasons, 2017 (same season as the transcriptome analysis, **Figures 5 A, C, E, G, I**) and 2018 (**Figures 5B, D, F, H, J**). The *ERF* gene (PRUPE_3G062800; **Figure 5A, B**) showed a downward trend of expression from Day 1 of storage in both cultivars, with a more rapid fall in SAG than in BT, in agreement with the transcriptome data where it was classed as an MD gene in SAG but not in BT. In 2018 expression of this gene also fell at later storage time points although the difference between the two cultivars was less clear. A second *ERF* gene (PRUPE_2G272500) also classed as MD in SAG but not BT (**Table 1**), fell in expression from day 5 in SAG (by almost 8 fold), and day 7 (by 4.5 fold) in BT in 2017 fruit, again in agreement with the transcriptome (**Figure 5C**). However, in 2018 expression in BT fell much more rapidly with storage compared to 2017 while expression of this gene in SAG was similar in trend to that in 2017 (**Figure 5D**). In contrast an *ACO* gene (PRUPE_3G209900), classed as an MI gene in BT but not SAG (**Table 1**), was upregulated during storage in both cultivars and both years, although in 2017 its expression peaked in SAG at day 7 and in BT at day 5, whereas in 2018 SAG expression continued to rise until day 14, and in BT it remained stable after day 5 (**Figures 5E, F**).

Two *AUX/IAA* genes (PRUPE_1G208300 and PRUPE_6G343800) were both down-regulated later in storage (**Figures 5G–J**) in both cultivars. PRUPE_1G208300 was classed as an MD expressed gene in SAG but not in BT while PRUPE_6G343800 was classed as MD expressed in both cultivars (**Table 2**). Expression of PRUPE_1G208300 in SAG fell from day 0 to day 5 (by 8 fold) in 2017 then rose again on day 7 but fell again by day 14, while in the 2018 season expression remained stable until day 1 then fell continuously between day 1 and day 14 (by 51 fold between Day 1 and Day 14). This gene in BT was not in the MI or MD expression groups and its expression did not show a clear trend in either year by real-time PCR. PRUPE_6G343800 expression fell with storage in both cultivars and both years at later time points, although the trend was clearer for BT. However, expression of this gene in BT only fell from day 0 in 2017 whereas it fell continuously from day 0 in the 2018 season.

### Expression of monotone genes involved in the cell wall, membrane and lipid modification

Of the MD and MI cluster genes in both cultivars, 32 were of relevance to CI (based on the literature), comprising genes related to cell wall metabolism, membrane structure and lipid metabolism (**Table 3**). In SAG, the expression of three polygalacturonase family proteins (PG), two polygalacturonase inhibiting protein 1 (PGI), and one beta-xylosidase 1, increased continuously, while four PGs, three pectin methylesterase inhibitor superfamily (PMEIS), and one expansin (Exp) gene were downregulated during the storage time course (**Table 3**). In BT, expression of one cellulase 2 (EGase 2), four PGs, the two PGIs, one PME, two PMEIS one pectinesterase, one beta-xylosidase 1, two Exp genes, and one EG45-like protein, increased continuously. Moreover, one PMEIS and two other Exp genes in BT were continuously downregulated across the time course (**Table 3**).

**Table 3:** Expression of monotone genes involved in the cell wall metabolism and membrane structure.

DESCRIPTION	ID	MI_SAG	MI_BT	MD_SAG	MD_BT
Cellulase, EGase, 2	PRUPE_5G131300				
glycoside hydrolase family 28 protein/polygalacturonase (pectinase) family protein (PG)	PRUPE_7G120900				
Pectin lyase-like superfamily protein (PL) Polygalacturonase (PG)	PRUPE_3G287200				
Pectin lyase-like superfamily protein (PL) Polygalacturonase (PG)	PRUPE_4G116600				
Pectin lyase-like superfamily protein (PL) Endo-polygalacturonase (PG)	PRUPE_4G262200				
Pectin lyase-like superfamily protein (PL) Polygalacturonase (PG)	PRUPE_1G110100				
Pectin lyase-like superfamily protein (PL) Polygalacturonase (PG)	PRUPE_1G129300				
Pectin lyase-like superfamily protein (PL) Polygalacturonase (PG)	PRUPE_2G175100				
Pectin lyase-like superfamily protein (PL) Polygalacturonase (PG)	PRUPE_2G301000				
Pectin lyase-like superfamily protein (PL) Polygalacturonase (PG)	PRUPE_7G269200				
Pectin lyase-like superfamily protein (PL) Polygalacturonase (PG)	PRUPE_8G265400				
polygalacturonase inhibiting protein 1 (PGI)	PRUPE_7G072600				
polygalacturonase inhibiting protein 1 (PGI)	PRUPE_7G072700				
pectine methylesterase (PME)	PRUPE_7G192800				
Plant invertase/pectin methylesterase inhibitor superfamily protein (PMEIS)	PRUPE_2G279700				
Plant invertase/pectin methylesterase inhibitor superfamily protein (PMEIS)	PRUPE_8G263900				
Plant invertase/pectin methylesterase inhibitor superfamily protein (PMEIS)	PRUPE_5G076800				
Plant invertase/pectin methylesterase inhibitor superfamily protein (PMEIS)	PRUPE_6G318500				
Plant invertase/pectin methylesterase inhibitor superfamily protein (PMEIS)	PRUPE_2G310600				
cell wall/vacuolar inhibitor of fructosidase 1/pectinaesterase	PRUPE_1G114500				
beta-xylosidase 1	PRUPE_1G123100				
Expansin (Exp)	PRUPE_2G136500				
Expansin (Exp) A1	PRUPE_5G195200				
Expansin (Exp) B2	PRUPE_2G274400				
expansin A8	PRUPE_6G042000				
Expansin (Exp)	PRUPE_8G174500				
EG45 PROTEIN	PRUPE_1G472300				
Peroxidase (POD)	PRUPE_4G021100				
LOX1, lipoxygenase	PRUPE_6G324100				
LOX2, lipoxygenase	PRUPE_2G005300				
orthologous to Arabidopsis FAD2	PRUPE_7G076500				
orthologous to Arabidopsis FAD8	PRUPE_6G056100				

Red indicates up-regulated and blue indicates down-regulated.

Five genes in these clusters were related to lipid metabolism (**Table 3**). Of the two lipoxygenase genes one (LOX1) was upregulated, but only in BT, while the other was downregulated in both cultivars (LOX2). A peroxidase (POD) gene was also downregulated, but only in BT, whereas the two FAD genes were both upregulated though only one of them in both cultivars.

A further 14 genes were related to stress and defence responses. These included four thaumatin, one dehydrin gene and one late embryogenesis abundant protein 25 (LEA-25/LEA-D113) (**Table 4**) in the MI class in both cultivars, Another two thaumatin genes were only upregulated in BT, and another LEA gene (LEA14) was upregulated in BT but down-regulated in SAG. Five heat shock genes were also represented in the BT monotone clusters, with mixed patterns of expression: three were upregulated, while the other two were downregulated.

**Table 4:** Expression of monotone genes related to pathogen and stress response.

DESCRIPTION	ID	MI_SAG	MI_BT	MD_SAG	MD_BT
Thaumatin	PRUPE_3G144100				
Thaumatin	PRUPE_3G143900				
Thaumatin	PRUPE_3G144000				
Thaumatin	PRUPE_3G148300				
Dehydrin (cold-regulated 47)	PRUPE_7G161100				
Late embryogenesis abundant protein, LEA-25/LEA-D113	PRUPE_7G076400				
Thaumatin	PRUPE_1G364000				
Thaumatin (osmotin)	PRUPE_5G094200				
Late embryogenesis abundant protein, LEA-14	PRUPE_2G319000				
Heat shock factor (HSF)-type, DNA-binding	PRUPE_7G056700				
Heat shock protein 70 family	PRUPE_7G107600				
Heat shock factor (HSF)-type, DNA-binding	PRUPE_8G234900				
Heat shock protein 70kD, C-terminal domain	PRUPE_6G079800				
Heat shock protein 70 family	PRUPE_7G265200				

Red indicates up-regulated and blue indicates down-regulated.

### Global differences in gene expression of transcription factor families during cold storage ripening

Altogether, 52 genes encoding TFs, were differentially expressed in SAG (5 MI, 42 MD, 3 TI, 2 TD), and 73 in BT (30 MI, 41 MD, 2TI). Most TF families in the MI and MD expression clusters were down-regulated in both cultivars. However, WRKY TFs were also upregulated in both cultivars and C2H2 family TFs were also upregulated in BT but not in SAG (**Table 5**). Relatively few TF families were transiently expressed, and many fewer TF families were amongst the MI class either cultivar compared to those in the MD expression class.

**Table 5:** Assignment of TF families to expression clusters.

DESCRIPTION	MI_SAG	MI_BT	MD_SAG	MD_BT	TI_SAG	TI_BT	TD_SAG	TD_BT
WRKY	1*	3	1					
bZIP	1	3	2	2				
NAC		1	3	3	1			
NF-YB			1	1				
bHLH	2	1	7	6				
Dof			5	2				
ERF		4	3	7	1	1		
DBB			1	1				
CO-like			1	3				
HD-ZIP		4	2	1				
C2H2		1	7	2				
C3H			1	1				
MYB	1	5		5		1		
G2-like			1	1				
GRAS			2		1			
GRF			1					
TALE			1	1				
ZF-HD			1					
HSF		2					2	
EIL				1				
MIKC_MADS				1				
SBP		1		1				
TCP				1				
MYB-RELATED			2	1				
GATA		1						
LBD		1						
Thrielix		1						
RAV		1						
B3		1						

*Number of genes in each family for each expression clusterRed indicates up-regulated and blue indicates down-regulated.

Only 16 TFs showed a differential expression pattern in both cultivars (**Table 6**). One WRKY gene (PRUPE_3G098100) was monotonically upregulated and 11 genes from other families were monotonically downregulated in both cultivars (**Table 6**). However, a C2H2-like zinc finger (PRUPE_3G048600) gene was monotonically upregulated in BT but downregulated in SAG and PRUPE_6G064700, an ERF TF also differed in expression between cultivars, with monotonic upregulation in BT and transient upregulation in SAG. Conversely, PRUPE_1G441700 (MYB) showed monotonic upregulation in SAG and transient upregulation in BT. Lastly, PRUPE_8G234900 (HSF), was monotonically upregulated in BT and transiently downregulated in SAG (**Table 6**).

**Table 6:** Expression pattern of TF genes that are found in the MI or MD clusters in at least one cultivar.

TF FAMILY	ID	MI_SAG	MI_BT	MD_SAG	MD_BT	TI_SAG	TD_SAG	TI_BT	TD_BT
bHLH	PRUPE_5G100700								
bZIP	PRUPE_1G419700								
C2H2	PRUPE_1G366300								
C2H2	PRUPE_3G048600								
C3H	PRUPE_1G416500								
CO-like	PRUPE_3G245100								
DBB	PRUPE_3G155900								
Dof	PRUPE_5G210200								
ERF	PRUPE_3G062800								
ERF	PRUPE_6G064700								
HD-ZIP	PRUPE_5G064300								
MYB	PRUPE_1G441700								
NAC	PRUPE_4G040900								
NF-YB	PRUPE_4G242700								
WRKY	PRUPE_3G098100								
HSF	PRUPE_8G234900								

Red indicates up-regulated and blue indicates down-regulated.

### Expression correlation between ethylene-associated genes and downstream CI-related genes

Weighted gene correlation network analysis (WGCNA) was applied to investigate correlations between patterns of expression of ethylene related genes (including biosynthesis, signalling and ERF transcription factors) and downstream genes involved in the cell wall and membrane composition modification, as well as pathogen and stress responses, which are relevant to CI symptom development.

**Figure 6:**
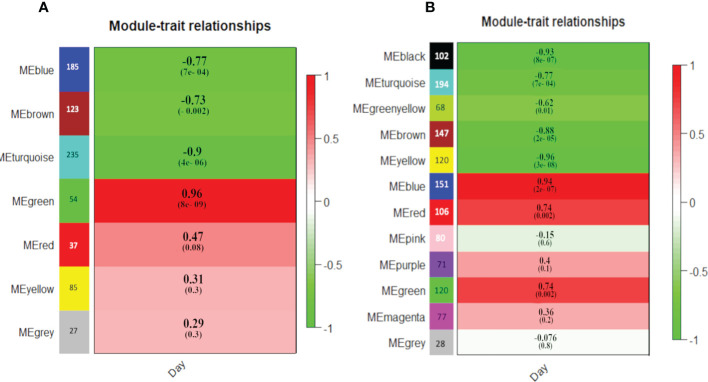
Module-Day trait association identified by WGCNA in SAG peach **(A)** and BT nectarine **(B)**. Each row corresponds to a module. The number of genes in each module is indicated on the left. The heat map indicates the correlation of each module with days of storage with the score and significance (P values in brackets) according to a Pearson analysis.

For SAG this analysis included all 746 DEGs which were grouped into seven co-expression modules (**Figure 6A**; **Supplementary Table 3**). Three modules (blue, brown and turquoise) were significantly and negatively correlated with “day of storage”, while one module (green) was significantly and positively to correlated storage. The largest significantly correlating module (turquoise) contained 235 genes, the smallest (green module) only contained 54 genes (**Figure 6A**).

Four SAG ERFs clustered into modules with significant correlation to “day of storage”. Three of these that were in the SAG MD gene expression cluster were included in modules showing a significant negative correlation with “day of storage” (two in the blue and one in the turquoise modules). The other *ERF*, also in the blue module showed TI expression. Amongst the 185 genes in the blue module, six were relevant to CI development or stress responses: a *MAPKKK* gene, one *Exp* and two PG genes, all of which showed an MD expression pattern in SAG, and an *HSF* which showed a TD trend (**Supplementary Table 4**). In the turquoise module, which included the other SAG MD-cluster *ERF*, potential downstream genes comprised a *LOX2* gene, two *PG*s and a *PMEIS* protein, with an overall MD expression pattern as well as two *PGI*s and a further *PG* gene, a *thaumatin* and a *dehydrin*, that surprisingly showed MI expression. None of the ERF genes were found in the brown and green modules, although both modules included genes related to CI-related downstream processes, as well as genes related to ethylene biosynthesis: an *ACS* in the green module and an *ACO* in the brown module (**Supplementary Table 4**).

WGCNA clustering of the 1264 DEGs for BT resulted in twelve distinct co-expression modules (**Figure 6B**; **Supplementary Table 5**). Five of them were significantly negatively correlated (black, turquoise, greenyellow, brown and yellow) while three modules were significantly positively correlated (blue, red and green modules) with storage days. Ten *ERF* genes and one *RAV* gene were clustered into these significant modules. An *ACS*, and two *ERF* genes were in positively correlated modules (blue and red) and were also MI expressed, while a third *ERF* gene was in the positively correlated green module but was an MD expressed gene. Co-expressed with these *ERF* genes were eleven MI expressed cell wall modification genes comprising four *PG*s, one *PGI*, one *PME* gene, one pectin esterase, two *Exp* genes, one *EG45* protein (all in the blue module), one *PMEIS* gene (red module), as well as one *FAD8*, related to lipid modification (blue module), also MI expressed. Five genes related to stress responses were also in the positively co-expressed modules: three thaumatin, and two *HSF* genes all MI expressed (**Supplementary Table 4**, blue, red and green modules).

Twelve BT genes related to ethylene metabolism and signalling were in the negatively co-expressed WGCNA modules. These comprised five *ERF* genes which were in the MD expression group (in yellow, greenyellow and brown modules) as well as an *EBF1/EIN3* and two *ERF* genes (black module) an *ACO* an *ETR2* and a *RAV* gene (yellow module) which were MI expressed (**Supplementary Table 4**). These correlated in their expression pattern with seven cell wall modification and three lipid modification genes in the same negatively co-expressed WGCNA modules: two *Exp*, one *PMEIS*, one *LOX2* and one *POD* gene, all MD expressed, as well as an *EGase cellulase*, one *beta-xylosidase*, one *PMEIS*, one *PGI* and *FAD2*, that were classed as MI expressed (across the black, brown, greenyellow and yellow modules). Six stress/pathogen-related genes were included in the significantly negatively correlated modules: two *HSF* genes that were MD expressed but also three *thaumatin* and one *LEA*, that were MI expressed despite being included in the negatively correlated modules.

### Promoter scanning analysis of downstream genes for ERF binding sites

To explore the potential of *ERF* and *RAV* and genes as regulators of downstream genes identified by WGCNA, their promoters were scanned for ERF binding sites. As expected, binding sites were not identified in the promoters of all co-expressed genes (**Supplementary Table 4**). However, in SAG five genes were identified as possible ERF targets including a *PG* and an *HSF* (TD) associated with three *ERF* genes, and a *PMEIS*, a *PG* and a *dehydrin* correlating with a different *ERF* gene. In BT fourteen downstream genes were associated with ten *ERF* genes and one *RAV* gene, including one *PG*, one *pectinesterase*, one *Exp*, one *EGase*, two *FADs*, two *thaumati*n, two *HSF*, three *PMEIS*, and a *POD* gene.

### PpERF and RAV in silico protein and phylogenetic analysis

**Figure 7:**
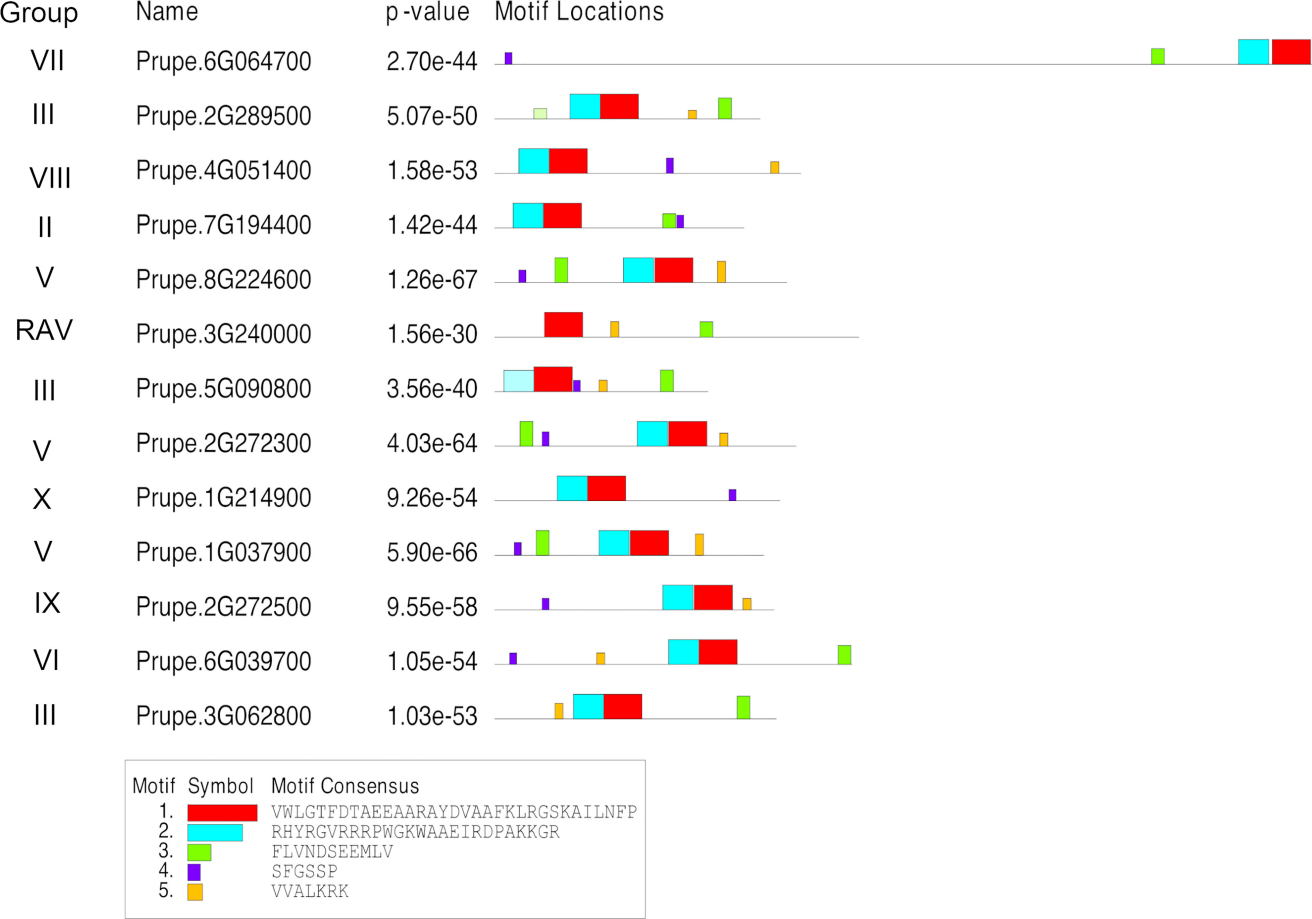
Gene structure and conserved motifs within thirteen peach ERF proteins identified through WGCNA as correlating in expression with downstream genes containing ERF binding motifs in their promoter sequences. Motifs identified using the MEME suite.

A new phylogenetic analysis of the ERF/RAV family was performed (based on the new peach genome sequence, Verde et al., 2017) to assign the 12 *ERF* genes and one *RAV* gene identified in WGCNA to the correct groups (**Supplementary Figure 5**; **Supplementary Table 6**). The new phylogeny identified 30 AP2 proteins (including 12 isoforms), 139 AP2/ERF proteins (including 16 isoforms) and seven AP2/RAV proteins (one isoform) in *Arabidopsis thaliana* while in peach, five *RAV* genes (eight, including isoforms), 19 *AP2* genes (6 isoforms) and 102 *ERF* genes (14 isoforms) were identified. This divided the *ERF* sub-family into eleven groups (I–X) where I to IV belong to the DREB family, and V to X, to the *ERF* family. Almost all of the eleven ERFs groups, were present in both SAG peach and BT nectarine fruit transcriptomes with Groups III and V being highly represented. The 13 *ERFs* identified from the WGCNA and promoter analysis were assigned to eight of these groups and one to the *RAV* subfamily (**Supplementary Table 6**; **Figure 7**).

### Motif and gene structures of *PpERF* genes

Motif structures of all the 13 *PpERF* and *ppRAV* genes identified in WGCNA modules with downstream genes (**Supplementary Table 4**), were analysed based on the presence of five conserved motifs (motifs 1–5) identified using the MEME suite (**Figure 7**). Motifs 1-2 that correspond to the AP2/ERF domain were identified in nearly all proteins analysed, except for the ppRAV protein PRUPE_3G240000 which lacks the second motif thus and thus appears to be divergent from the rest. Motifs 3-5 also discriminated the proteins: three *PpERFs* (PRUPE_4G051400, PRUPE_1G214900 and PRUPE_2G272500) lacked the third motif, three other *PpERF* genes (PRUPE_2G289500, PRUPE_3G240000, and PRUPE_36062800) lacked the fourth motif and the fifth motif was absent in three other *PpERFs* (PRUPE_6G064700, PRUPE_7G194400 and PRUPE_1G214900) (**Figure 7**). All the five motifs were thus present in only five ERF proteins analysed: PRUPE_6G039700, PRUPE_2G272300, PRUPE_8G224600, PRUPE_5G090800, PRUPE_1G037900 (**Figure 7**).

## Discussion

### BT nectarine responds earlier and more strongly to cold storage stress

Under cold storage, fruits experience stress, and as is found in whole plants, their tolerance differs between species and cultivars, and is reflected by a differential transcriptional modulation (Haak et al., 2017). In cold-sensitive peach, CI is one of the most seriously damaging effects of cold storage, and both genomic and transcriptome studies have been performed to identify the genetic pathways responsible for the metabolic disorder which causes the injury (e.g. Ogundiwin et al., 2008; Cantín et al., 2010; Lurie, 2021). However, relatively few studies have investigated molecular mechanisms triggered in response to chilling stress, before CI develops, during a storage time-course (Pons et al., 2014; Puig et al., 2015; Pons et al., 2016; Wang et al., 2017).

Data presented here showed clear changes in transcriptomic profiles of both cultivars, with a prevalence of genes that were classed as “monotone” i.e., whose expression changed continuously and in the same direction during the time course. Moreover, most of the changes occurred after only 1 and 5 days of storage, indicating that most responses to the cold stress occurred early in the storage period. This highlights the importance of assessing the effects of cold storage at very early time points as well as the longer intervals used in several other studies (e.g. Vizoso et al., 2009; Pons et al., 2014; Puig et al., 2015; Pons et al., 2016; Nilo-Poyanco et al., 2019). There were almost twice as many DEGs in BT (1264 DEGs) compared to SAG (746 DEGs), and the most changes were found between day 1 and day 0 suggesting that BT nectarine fruit responded very rapidly to the cold while SAG peach responded more slowly. This could be linked to the higher ripening index at harvest of the BT fruit, also linked to differences in the later development of CI (Lurie and Crisosto, 2005) or could be a varietal difference. Nectarines are often more resistant to CI than peaches (Dagar et al., 2011; Giné-Bordonaba et al., 2016), and BT nectarine was previously shown to develop less CI symptoms than another cultivar, ‘Venus’ (Cantín et al., 2010). The higher level of transcriptomic responses of BT nectarine at these early time points, well before any symptoms of CI are evident, could therefore be related to chilling tolerance later during storage although more cultivars would need to be assessed together with the timing and specific CI symptom development ion each cultivar.

### Cold storage affects the monotone DEGs differentially in the two cultivars

Although there was a prevalence of genes continuously down- or upregulated for both cultivars in response to cold exposure, a higher proportion (62%) of SAG peach genes were down-regulated (MD class) compared to BT nectarine (50%). Differences in global down-regulation of gene expression amongst cultivars has been noted previously (Puig et al., 2015), although in this study the first timepoint assessed was at 7 days. Here similar types of genes and pathways were expressed differentially in both SAG and BT in response to cold. Downregulated genes were consistently linked to photosynthesis. This could be due to a combination of the ripening that continues during storage and during which chloroplasts are converted to chromoplasts (Chen et al., 2018) and chloroplast damage during chilling. Indeed, in tomato fruit, cold storage resulted in downregulation of chloroplastic ATP synthase which was interpreted as a response to damage to chloroplasts rather than a direct cold response (Sánchez et al., 2012). In contrast, upregulated genes in both SAG and BT were more associated with stress response, defence mechanisms, and metabolic pathways in line with other studies (Falara et al., 2011).

In both cultivars, ethylene and auxin pathways, which are essential for normal peach ripening (Trainotti et al., 2007; Tatsuki et al., 2013) were affected by cold storage. The greater downregulation of several *ERF* genes in SAG compared to BT at most timepoints from real time PCR data is consistent with the higher ethylene production in BT at 14 days, although the *ACO* expression differences were not consistent: this may be due to action of other *ACO* gene family members or post-transcriptional regulation. During prolonged cold storage, maintaining the ability of nectarine fruit to produce ethylene or adding exogenous ethylene to the storage atmosphere, prevents CI (Zhou et al., 2001). Moreover, both gene expression and protein levels of ACO and ACS1 were depleted during cold storage in fruit developing CI (Dong et al., 2001; Zhou et al., 2001). The results here, therefore, are consistent with BT nectarine retaining ethylene signalling for longer and may be important at later storage timepoints in reducing the development of CI.

In contrast, changes in auxin related pathway genes did not show a clear trend, although there appeared to be a slightly greater downregulation of pathways related to auxin synthesis and transport in SAG peach compared to BT nectarine. Subcellular accumulation of auxin and auxin signalling were linked to chilling responses in peach fruit (Puig et al., 2015). Here, genetic pathways related to auxin downstream signalling did not show clear intercultivar differences: a comparable number of auxin-responsive genes were either down or upregulated in both SAG and BT. However, auxin initiates ripening by inducing system II ethylene production (Puig et al., 2015), therefore, auxin here may be working synergistically with ethylene in modulating chilling responses.

Clear inter-cultivar transcriptomic differences under cold storage were found for genes related to cell wall metabolism with a greater number of *PG* and *PMEIS* genes upregulated in BT compared to SAG and downregulated in SAG compared to BT. This may play a role in the more rapid loss of firmness in SAG compared to BT. Up-regulation of these genes is also consistent with susceptibility of peaches to developing CI during long-term cold storage since these changes in expression can be linked to cell wall metabolic disorders (Lurie and Crisosto, 2005). However, although similar genes were also previously reported as changing in expression during storage of peach fruit (Wang et al., 2017) there were differences in the specific changes which may reflect the different cultivars studied, differences in time points or initial stage of maturity.

Long term cold exposure reduces membrane fluidity, mainly through the modulation of genes involved in lipid metabolism and can eventually result in internal browning (Routaboul et al., 2000; Browse and Xin, 2001; Martz et al., 2006). In peach, high expression levels of *PpLOX* and genes related to proanthocyanin monomer biosynthesis were associated with browning sensitivity (Peace et al., 2005; Ogundiwin et al., 2008; Puig et al., 2015). Internal browning was not detected in either cultivar here even after 3 weeks of cold storage. The absence/downregulation of both *PPO* and *POD* gene expression, together with a continuous downregulation of *LOX2*, which would negatively affect membrane permeability, may provide a possible explanation. Moreover, we also detected an upregulation of *FAD8* in both SAG and BT and *FAD2* only in BT. These genes encode membrane desaturase enzymes, which would lead to greater membrane fluidity by increasing the amount of unsaturated fatty acids, thus contributing to maintaining cell integrity. These changes in gene expression during the first 2 weeks of storage studied here may be important in delaying CI.

PR genes were well represented among the DEGs including genes encoding thaumatin-like proteins, LEA proteins and heat shock proteins. Accumulation of thaumatin-like protein as a cold response in peach fruit occurred earlier in fruit cell walls of a CI-resistant cultivar (Dagar et al., 2010). Thaumatin-like proteins have a role in cryoprotection (Liu et al., 2010) and may be important in counteracting the alterations in cell wall structure that characterize the onset of CI, leading to woolliness. Some of the PR genes were exclusively differentially expressed in BT nectarine, and the number of upregulated PR genes was greater in BT nectarine than in SAG, showing a differential response to cold in the two cultivars.

### Co-expression analysis of DEGs containing PpERF binding motifs identifies new potential regulators of lipid and cell wall metabolism during chilling.

The recent re-annotation of the peach genome (Verde et al., 2017) resulted in reallocation of some of the *AP2/ERFs* to different classes based on phylogeny, especially in the groups I, II, V, VI and VIII, which in turn showed a higher number of annotated proteins compared to previous annotation.

Co-expression of *ERFs* with cell wall and lipid metabolism genes had already been noted (Wang et al., 2017). Of the 32 ERFs identified previously as potentially involved in peach fruit postharvest chilling responses (Wang et al., 2017) four were identified amongst the 13 *ERF* genes that were co-expressed with downstream chilling response processes here. Amongst these 13 *ERF* genes, motif analysis showed that expression of *ERF* genes with four motifs (**Figure 6**), seemed to be better correlated with downstream genes involved in regulation of cell wall and membrane modulation (**Supplementary Table 4**); while *ERF* genes with five motifs (**Figure 6**) correlated more closely with genes involved in pathogen and stress response (**Supplementary Table 4**).

One of these is PRUPE_2G272500 (previously ppa012014m and denoted *ERF2* in Wang et al., 2017), classed here as a Group IX ERF. Although Arabidopsis *ERF2* also belongs to this group, Arabidopsis *ERF106* and *ERF107* also known as *DEWAX2* and *DEWAX* respectively are much closer to PRUPE_2G272500 in the new phylogenetic tree. *DEWAX2* was previously shown to negatively regulate cuticular wax biosynthesis in Arabidopsis leaves (Kim et al., 2018). PRUPE_2G272500 was co-expressed here in SAG peach with two other *ERF*s one also in Group VII (PRUPE_6G064700) and the other in Group VI (PRUPE_6G039700). Amongst their co-regulated downstream genes, a *PG* and an *HSF* gene are plausible targets, as they contain *ERF* binding sites in their promoters. PRUPE_6G064700 had not previously been annotated as an AP2/ERF gene and hence may be a new potential regulator of downstream CI-related processes. Regulation of cell wall modulating genes and stress responsive genes has been noted for Group IX *ERF*s in other species e.g., in grape berry development (Deluc et al., 2007). Expression of other *ERF*s, belonging to Groups III (PRUPE_5G090800) and Group V (PRUPE_2G272300), also correlated in this study with *HSF* expression in the BT nectarine. Interactions between ERFs and HSFs has been previously reported in other systems. For example, in sunflower seeds, a DREB TF enhanced the action of an HSF on seed longevity (Almoguera et al., 2009). Hence it is possible that ERF-HSF interaction has a role in peach fruit responses to chilling. Given that different *ERF* genes are associated with *HSF* expression in BT and SAG this may also reflect different responses of the two cultivars to cold storage

Wang et al. (2017) did not place *ERF2* in a co-expression network with cell wall related genes, which may reflect different algorithms used for co-expression analysis, or perhaps differences in the chilling regimes imposed or cultivar studied. However, under constant 0°C chilled storage *ERF2* expression peaked at 5 days and then fell back (Wang et al., 2017), which is not dissimilar to its expression in BT nectarine seen here in both transcriptome and real time PCR analyses, although in SAG peach expression peaked earlier at day 1. Under constant 0°C chilled storage, one of the other *ERF*s identified here as a potential regulator of a *PG* and *HSF* gene was PRUPE_6G039700 (previously ppa025495m, denoted by Wang et al., 2017 as CRF4.1). This gene also showed an MD expression pattern here, and in the first 14 days of storage of the Wang et al. (2017) study showed a very similar pattern of change to *ERF2* (PRUPE_2G272500) although it fell into a different expression class. Our new phylogeny confirms the putative homology to Arabidopsis *CRF4* (*CYTOKININ RESPONSE FACTOR 4*) although the branch support is a little weak. The third gene in the co-expression module identified here, PRUPE_6G064700, was not previously annotated as an *AP2/ERF* gene in Zhang et al. (2012), and hence is a new potential regulator of cell wall metabolism in peach under chilled storage. It is classed as a Group VII in the new phylogeny and its two closest Arabidopsis genes in our phylogeny are *ERF71* (*HRE2*) and *ERF72* (*RAP2.3*). *ERF71* seems to have multiple roles in Arabidopsis hypoxia (Eysholdt-Derzsó and Sauter, 2018) and pathogen, response signalling (Yelli et al., 2018); *ERF72* in Arabidopsis is thought to control NO homeostasis linked to ABA and JA (Léon et al., 2020). In addition, Group VII *ERF*s have also been associated with the regulation of ripening and senescence in fruit including plum and apple (Tournier et al., 2003; Wang et al., 2007; El-Sharkawy et al., 2009.) Hence a role for this peach gene in stress signalling is plausible, although it might also have a role in the continued ripening during the storage period.

PRUPE_2G272300 (previously ppa010186m, denoted by Wang et al., 2017 as ERF13) was upregulated both by storage at 0°C (Wang et al., 2017) and in this study. Wang et al. (2017) showed that it was part of gene networks regulating both lipid and cell wall metabolism specifically with an expansin, a pectin methyl esterase and a gene involved in sphingolipid biosynthesis. Here expression of PRUPE_2G272300 only correlated with expression of an HSF gene in BT nectarine. This suggests multiple roles for this gene both directly in the regulation of downstream pathways but also in stress regulation *via* an interaction with HSFs. However, in the new phylogeny it is not possible to infer functional roles from Arabidopsis since this gene is placed on a separate branch of the tree.

The fourth *ERF* gene identified both here and in Wang et al. (2017) as a DEG is PRUPE_8G224600 (ppa023839m, denoted in Wang et al., 2017, as *ERF1B.1*), a Group V *ERF*. In the new phylogeny this gene is closest to Arabidopsis *ERF15* and *ERF59* although branch support in this part of the tree varies widely. *ERF15* is a positive regulator of ABA responses in Arabidopsis (Lee et al., 2015), while *ERF59* has a role in SA responses (Caarls et al., 2017). Wang et al. (2017) also shows co-expression networks involving this ERF in both lipid and cell wall pathway control, however in this study, although its expression correlated with three downstream genes in BT nectarine, none of these genes had ERF binding sites in their promoter sequences. Thus, its role in chilling responses cannot be confirmed.

In this study, of the 13 *ERFs* co-expressed with genes related to downstream chilling response genes, three were assigned to Group III in the new phylogeny (PRUPE_2G289500, PRUPE_3G062800 and PRUPE_5G090800 already discussed above). In SAG peach co-expression of Group III ERFs was with cell wall and stress response genes, in BT nectarine co-expression was also detected with genes involved in lipid metabolism and membrane modulation. This is consistent with data from other plants where Group III ERFs are involved in stress and pathogen responses (Xu et al., 2008; Xie et al., 2016). One Group III ERF was also shown to activate a *PG* gene in apple and may be responding both to cold and ethylene signalling (Tacken et al., 2010). Thus, the group III *ERFs* in SAG peach and BT nectarine may be responding both to the ethylene produced by ripening during the storage and/or to cold signals.

Of the remaining 10 ERFs not discussed above, PRUPE_1G214900 was co-expressed with both cell wall and lipid metabolism but also with stress responses. In the new phylogeny, this Group X ERF is closest to Arabidopsis *ERF114*, *ERF115* and *RAP2.6L* genes, again though the branch support is not strong. All three of these genes in Arabidopsis respond to wounding (Ikeuchi et al., 2017) and may have a role in JA signalling. Since JA signalling is also important in plants’ response to cold (Hu et al., 2017) it is possible that PRUPE_1G214900 may be responding to the cold signals *via* JA and activating response genes in multiple pathways in BT nectarine. PRUPE_4G051400, a Group VIII ERF was also not identified by Wang et al. (2017) as involved in peach chilled storage responses. Group VIII ERFs are negative regulators of ethylene-, jasmonate-, and ABA-responsive genes (Nakano et al., 2006), also expressed in fruit (Qi et al., 2016). PRUPE_4G051400 is closest to *ERF9* in Arabidopsis which is a transcriptional repressor involved in responses to osmotic stress (Van den Broeck et al., 2017) and pathogens (Maruyama et al., 2013). Its putative downstream genes in BT nectarine are a *PMEIS* and a *POD;* these genes may therefore be repressed, perhaps to delay cold-induced texture changes. PRUPE_7G194400 (Group II) and PRUPE_3G240000 (RAV) are part of the same co-expression module. In the new phylogenetic tree, PRUPE_7G194400 is closest to Arabidopsis *ERF018* which in Arabidopsis is involved in JA signalling and binds to the promoter of lipoxygenase gene (Pauwels et al., 2008). However, the branch support is quite low (31%) and Arabidopsis *ERF017* which is also involved in JA signalling (Winter et al., 2007) is also part of this cluster on the tree.

In conclusion, early transcriptomic responses to chilling are detectable well before signs of CI and vary between cultivars. Common responses between the two cultivars indicate conserved responses to the cold treatment which may be widespread across different cultivars. Differences across the two cultivars may reflect different response mechanisms to the cold storage stress. Genes that are activated early may provide markers for detecting CI development before it can be detected visually, although further work would be needed across a wider range of peach cultivars correlating gene expression changes to CI resilience and assessing common and cultivar-specific responses. *ERF* gene expression correlated closely with expression of downstream processes known to be implicated in CI development. This suggests that ERF regulation may be important in CI development at a very early stage during cold storage. Verification of the direct regulation of downstream genes by the ERFs is needed as well as further functional analysis of ERF regulation across a wide range of cultivars. This may provide useful markers for breeding peaches and nectarines that are more resilient to chilling and therefore suitable for longer shipping routes.

## Data availability statement

The datasets generated for this study can be found in the Sequence Read Archive (SRA) database at NCBI (SRA BioProject PRJNA798864).

## Author contributions

AM, LB, MM, RL, MF, LS, CL, EP, LP, CM, AC, HR, MB and NS, conducted the experimental work and data analysis, AM, HR, and NS drafted the manuscript, AF, CM, HR, MB and NS designed the project. LB, AF, HR, MB, NS acquired the funding. All authors contributed to the article and approved the submitted version

## Funding

This research was supported by Fondazione con il Sud, call Brain2South, as part of the FRUITY collaborative project (2015-0245). N.D.S also benefits from funding of the program PON “Research and Innovation” 2014-2020 (PON R&I), Action IV.6 “Contratti di ricerca su tematiche Green”.

## Acknowledgments

We thank Campo Verde S.p.A. for providing fruit and refrigeration facilities.

## Conflict of interest

The authors declare that the research was conducted in the absence of any commercial or financial relationships that could be construed as a potential conflict of interest.

## Publisher’s note

All claims expressed in this article are solely those of the authors and do not necessarily represent those of their affiliated organizations, or those of the publisher, the editors and the reviewers. Any product that may be evaluated in this article, or claim that may be made by its manufacturer, is not guaranteed or endorsed by the publisher.
