# Supplementary material for: Comparative transcriptomic profiling of peach and nectarine cultivars reveals cultivar-specific responses to chilled postharvest storage
Source: Front Plant Sci. 2022 Nov 25;13:1062194. doi: 10.3389/fpls.2022.1062194 (PMC9733835; doi:10.3389/fpls.2022.1062194)
Supplement: Supplementary file 1 [file DataSheet_1.docx]

**
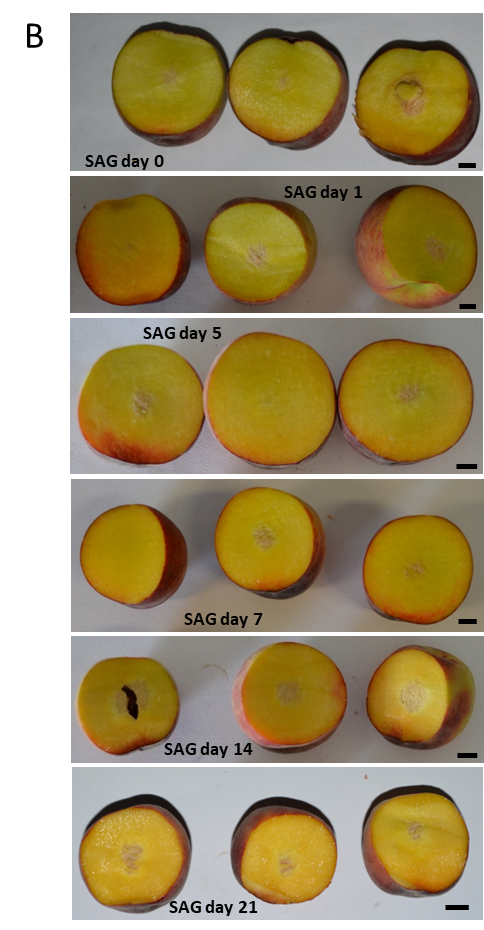

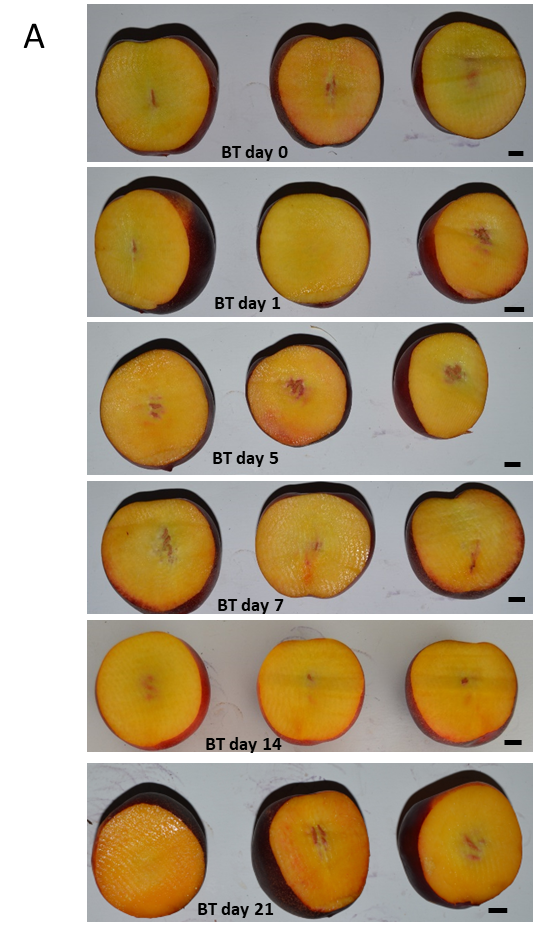
SUPPLEMENTARY FIGURES**

**Supplementary Fig 1.** Images of halved peaches from the two cultivars during storage: ‘Big Top’, BT (A) and ‘Sagittaria’, SAG (B) at 0, 1, 5, 7, and 14 days of storage at 1 ºC + 36 h recovery at ambient temperature (22 °C).


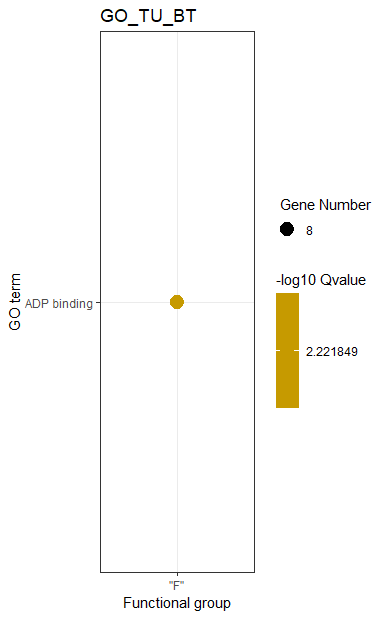

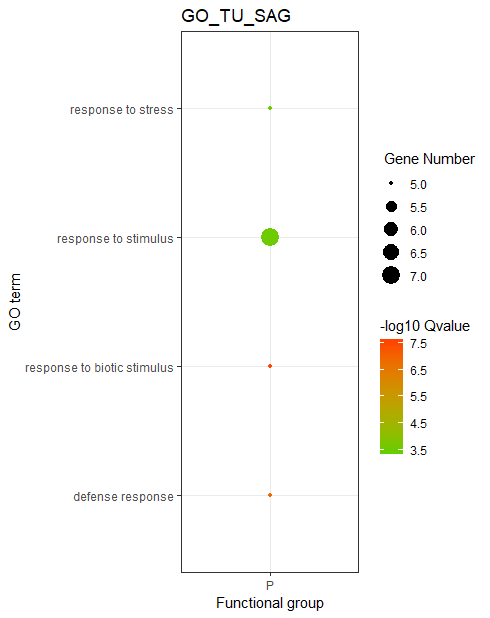


GO_TI_BT

GO_TI_SAG

A

B

**Supplementary Fig. 2.** GO enrichment analysis of TI cluster genes in the two cultivars: SAG (27) (A) and BT (62) (B). The pathway enrichment analysis was performed with KOBASS, online tool, and the detailed information is presented as a bubble chart, P = biological processes and F = molecular functions. The size of the bubbles represents the number of assigned genes, and the color of bubbles represents the ‑log10 (Q‑value).


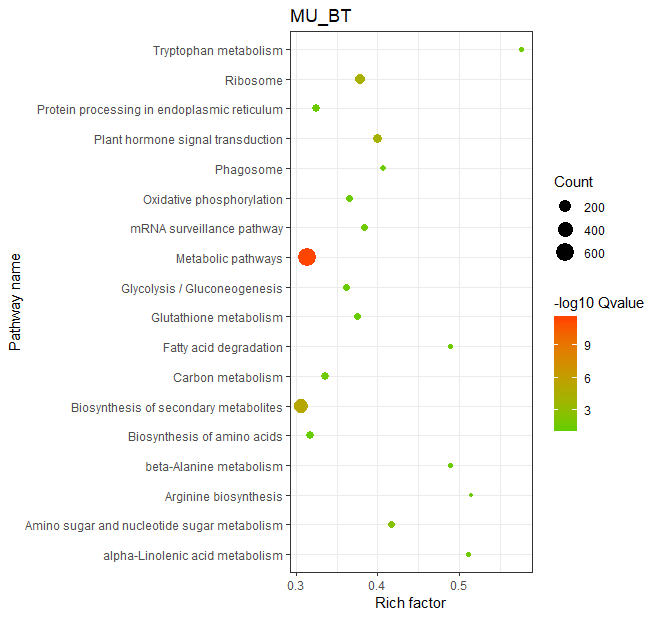

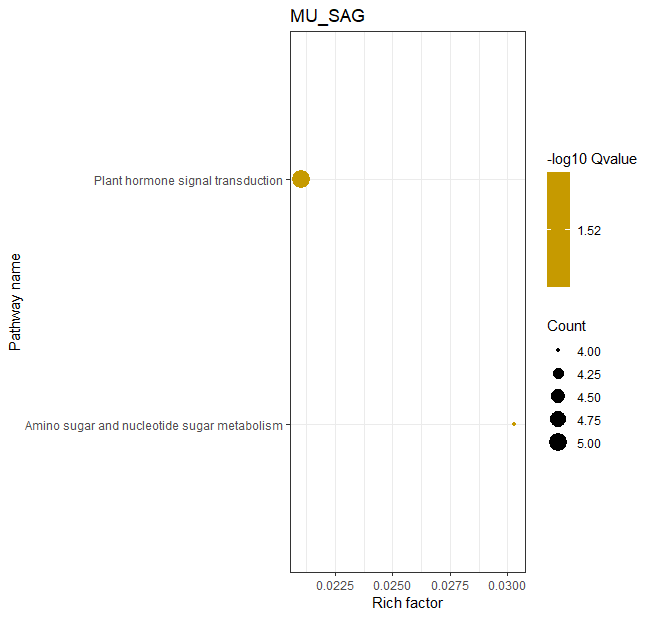


KEGG_MI_BT

KEGG_MI_SAG

B

A

D

C

KEGG_MD_SAG

KEGG_MD_BT


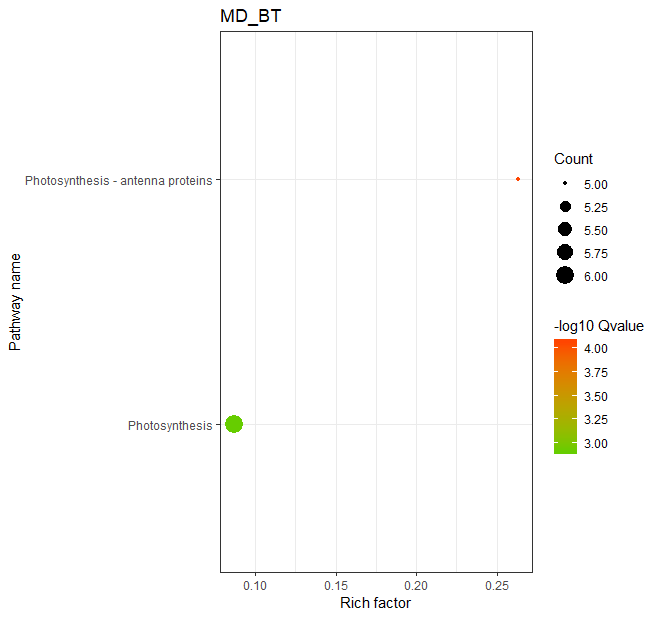

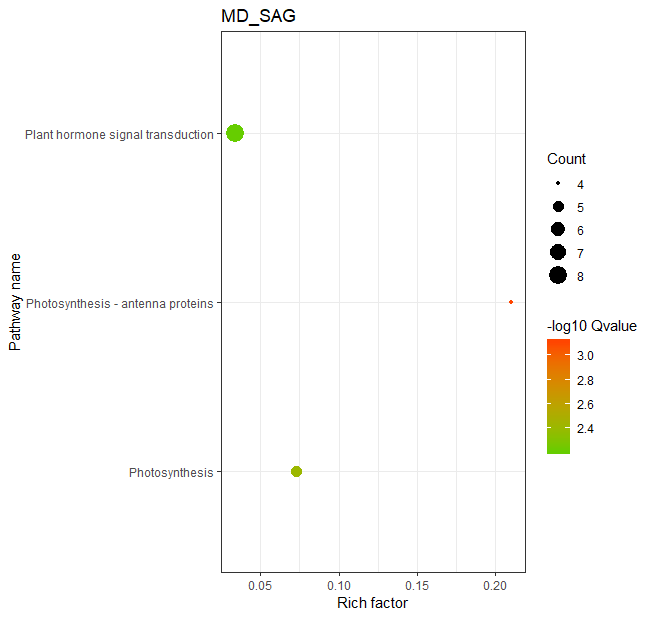


**Supplementary Fig. 3.** Pathway enrichment analysis for MI and MD cluster genes in the two cultivars: MI SAG (174) (A) and MI BT (468) (B); MD SAG (403) (C) and MD BT (456) (D). The pathway enrichment analysis was performed with KOBASS, an online tool, and the detailed information is presented as a bubble chart. The y‑axis represents the significantly enriched pathways, the x‑axis represents the richness factor, the size of the bubbles represents the number of assigned genes, and the color of bubbles represents the ‑log10 (Q‑value). The larger the number of genes classified into the pathway, the larger the node size. The more significant the pathway, the higher on the color bar the bubble is. KEGG, Kyoto Encyclopedia of Genes and Genomes; Q‑value, Bonferroni‑adjusted P‑value.

B

A


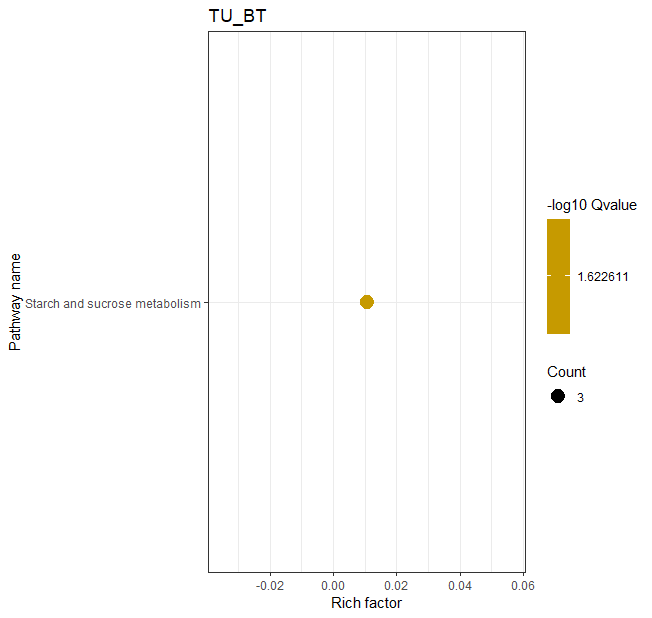

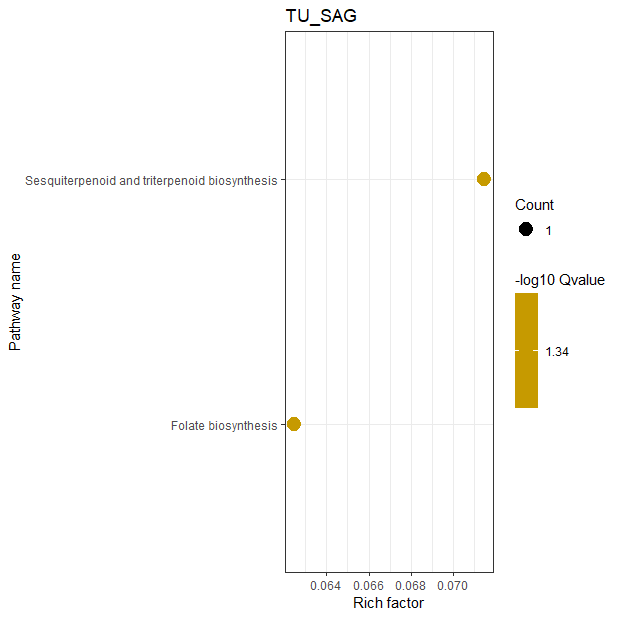


TI_BT

TI_SAG

**Supplementary Fig. 4.** Pathway enrichment analysis of TI cluster genes in the two cultivars: SAG (27) (A) and BT (62) (B). The pathway enrichment analysis was performed with KOBASS, an online tool, and the detailed information is presented as a bubble chart. The y‑axis represents the significantly enriched pathways, the x‑axis represents the richness factor, the size of the bubbles represents the number of assigned genes, and the color of bubbles represents the ‑log10 (Q‑value). The larger the number of genes classified into the pathway, the larger the node size. The more significant the pathway, the higher on the color bar the bubble is.


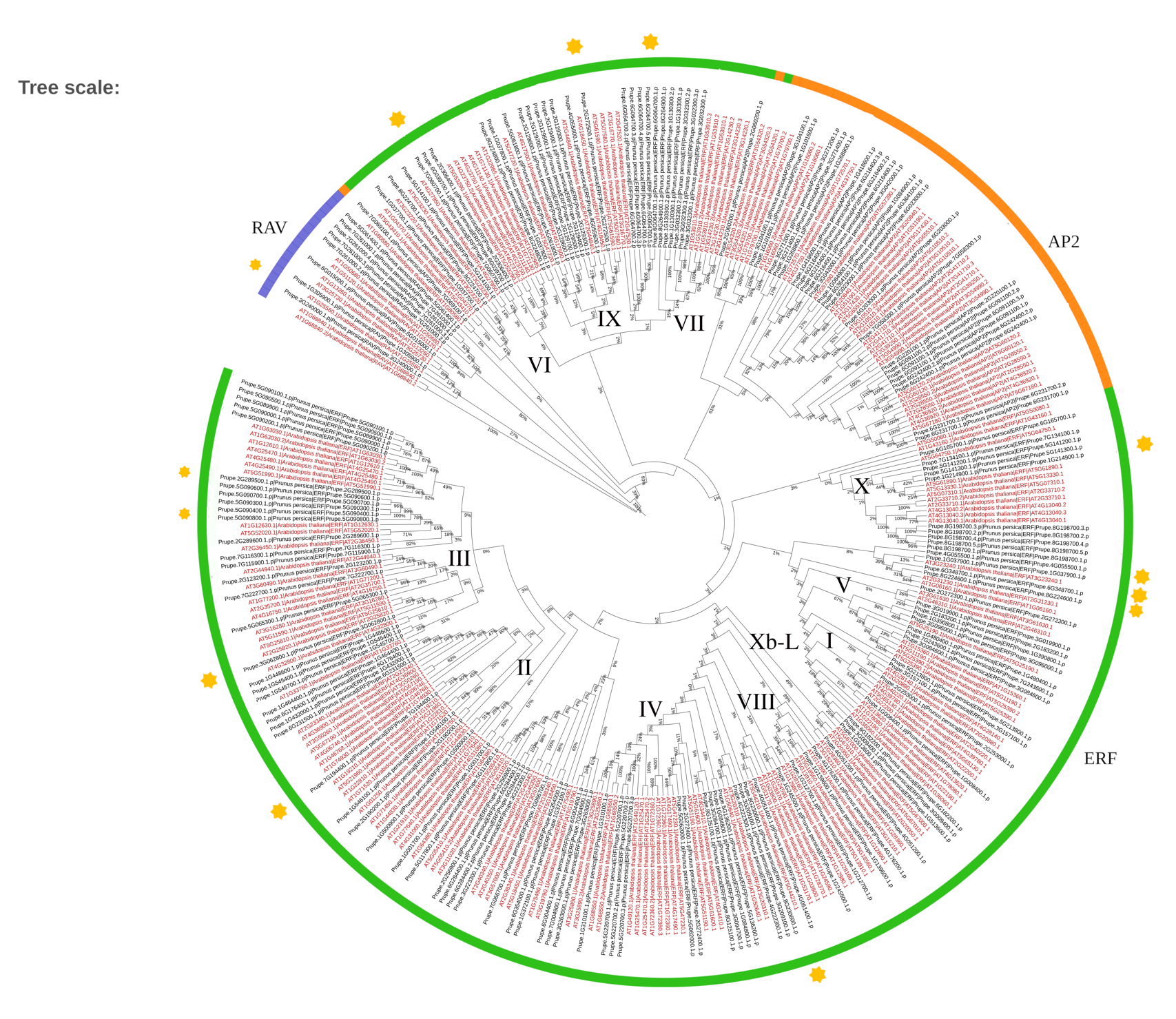


**Supplementary Fig. 5.** Phylogenetic tree of the AP2/ERF (APETALA2/ethylene-responsive factor) superfamily protein in peach and *Arabidopsis*. The tree was created by the bootstrap option of the CLUSTAL W multiple alignment packages and the neighbour-joining method using the 138 Arabidopsis AP2/ERF amino acid sequences (in red writing )and 131 peach AP2/ ERF amino acid sequences (in the black writing). The orange dot indicate the ERF reported in Fig. 7
